# Neuroprotection in metabolic syndrome by environmental enrichment. A lifespan perspective

**DOI:** 10.3389/fnins.2023.1214468

**Published:** 2023-08-10

**Authors:** Tamara Kobiec, Claudia Mardaraz, Nicolás Toro-Urrego, Rodolfo Kölliker-Frers, Francisco Capani, Matilde Otero-Losada

**Affiliations:** ^1^Facultad de Psicología, Centro de Investigaciones en Psicología y Psicopedagogía, Pontificia Universidad Católica Argentina, Buenos Aires, Argentina; ^2^Centro de Altos Estudios en Ciencias Humanas y de la Salud, Universidad Abierta Interamericana, Consejo Nacional de Investigaciones Científicas y Técnicas, Buenos Aires, Argentina; ^3^Facultad de Ciencias de la Salud, Instituto de Ciencias Biomédicas, Universidad Autónoma de Chile, Santiago, Chile

**Keywords:** metabolic syndrome, environmental enrichment, neuroprotection, neurodevelopment, lifespan, healthspan

## Abstract

Metabolic syndrome (MetS) is defined by the concurrence of different metabolic conditions: obesity, hypertension, dyslipidemia, and hyperglycemia. Its incidence has been increasingly rising over the past decades and has become a global health problem. MetS has deleterious consequences on the central nervous system (CNS) and neurological development. MetS can last several years or be lifelong, affecting the CNS in different ways and treatments can help manage condition, though there is no known cure. The early childhood years are extremely important in neurodevelopment, which extends beyond, encompassing a lifetime. Neuroplastic changes take place all life through — childhood, adolescence, adulthood, and old age — are highly sensitive to environmental input. Environmental factors have an important role in the etiopathogenesis and treatment of MetS, so environmental enrichment (EE) stands as a promising non-invasive therapeutic approach. While the EE paradigm has been designed for animal housing, its principles can be and actually are applied in cognitive, sensory, social, and physical stimulation programs for humans. Here, we briefly review the central milestones in neurodevelopment at each life stage, along with the research studies carried out on how MetS affects neurodevelopment at each life stage and the contributions that EE models can provide to improve health over the lifespan.

## Introduction

The term “metabolic syndrome (MetS)” was coined by Hans Haller in 1975 to refer to the coexistence of hyperglycemia, dyslipidemia, obesity, hepatic steatosis, and hypertension (Haller and Hanefeld, 1975). Yet, it was not until 1988, thanks to Reaven’s description of insulin-dependent glucose uptake resistance, v. g., insulin resistance, as the “syndrome X” that it became well-known (Reaven, 1988).

The first official definition of MetS was coined by a World Health Organization (WHO) Working Group in 1999, and various alternative definitions have followed from then on. The most widely accepted ones have been proposed by the National Cholesterol Education Program (NCEP) ATP III, the European Group for the Study of Insulin Resistance (EGIR), and the International Diabetes Federation (IDF). Regardless some differences, all of them include obesity (in the WHO definition BMI is referred to, while the others refer to central obesity, so they express this value as waist circumference in centimeters), high triglycerides or dyslipidemia, and hypertension (systolic at predominance). The ATP III, EGIR, and IDF definitions also include fasting plasma glucose, whereas the WHO definition includes microalbuminuria. Low HDL-cholesterol levels are present in the ATP III and IDF definitions. Two of these features (or three in the ATP III definition) are enough to diagnose MetS in adults (Balkau and Charles, 1999; World Health Organization, 1999; National Cholesterol Education Program, 2002; International Diabetes Federation, 2005).

The incidence of MetS has increased over the past years and has become a global health problem. In Western societies, its prevalence is around 23–35%. Obesity and diabetes — risk factors for MetS — have been increasing regardless the age (Schwarz et al., 2017; Chan et al., 2021). Also, the prevalence of dementia and cognitive dysfunction has been dramatically increasing, and many studies show the association between these conditions and MetS (Neergaard et al., 2017). In this sense, MetS has proved to produce neurological sequelae like cognitive impairment, risk of stroke, dementia, Alzheimer’s disease, and neurodegeneration, among others. However, the relationship between MetS and SNC still remains unclear in several aspects (Rashid et al., 2021; Więckowska-Gacek et al., 2021).

**Figure 1:**
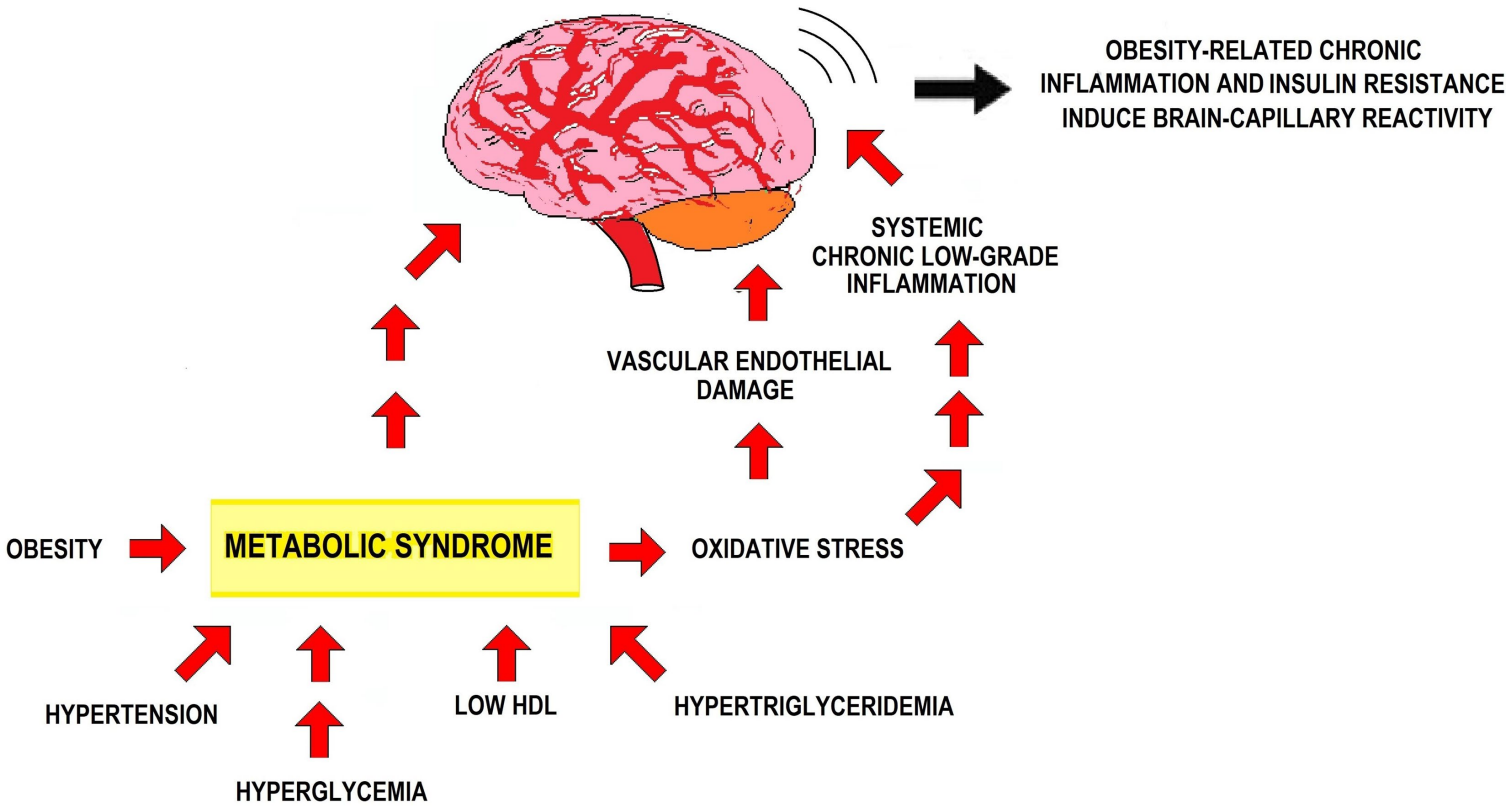
Effects of MetS on the CNS. The figure depicts how MetS’ clinical features affect the CNS, in particular via insulin resistance, oxidative stress, vascular endothelial damage, and inflammation.

MetS features favor capillary stiffness and thickness. This affects the carotid artery — the major blood supply to the CNS — reducing blood flow to the brain, which neither gets enough oxygen supply nor eliminates waste as much as needed. The resulting conditions for neurodevelopment are not optimal and lead to dysfunctions. Brain dysfunction due to endothelial changes goes together with increased oxidative stress and chronic inflammation. The core constituents of MetS start a perpetual cycle of events within the vascular wall, starting with endothelial dysfunction and progressing to oxidative stress, low-grade inflammation, and platelet hyperactivity. Insulin resistance and oxidative stress in Mets lead to vascular endothelial damage and persistent low-grade inflammation. The precise mechanisms linking MetS with neurodegenerative diseases remain uncertain. Yet, concurrent insulin resistance, vascular endothelial damage, oxidative stress, and low-grade inflammation shape a pathophysiological milieu that fosters brain degenerative conditions development (Yates et al., 2012; Han and Lean, 2015; Arshad et al., 2018; Lemieux and Després, 2020; Chan et al., 2021) (Figure 1).

MetS has a multifactorial and complex etiology, under both genetic and environmental influence, such as sedentary lifestyle, unhealthy habits, high fat and carbohydrate diet, family history, genetic predisposition, education, socioeconomics, among others. However, in the last years, it has been shown that genetic factors have less impact than environmental ones, and can be controlled by a healthy lifestyle (Chan et al., 2021).

One of the most important challenges regarding brain damage is that MetS individual conditions not only act separately but also synergistically, increasing MetS consequences on the Central Nervous System (CNS). For example, endothelial dysfunction could be caused by the concurrence of blood hypertension and chronic inflammation associated with obesity (Drożdż et al., 2022). However, neuroplastic changes occur throughout life, so the relationship between the brain and behavior must be considered throughout the life cycle. Along with genetic factors that exert their influence, the epigenetic response to environmental and internal changes — epigenetic events — affects the brain. Though the early childhood years are crucial in neurodevelopment, neuroplastic modifications continue beyond this stage, encompassing a lifetime. In adolescence, adulthood, and old age, neurodevelopmental changes continue ongoing, in response to internal and external — environmental — factors (Tucker-Drob, 2019; Queen et al., 2020).

Environmental enrichment (EE) is an animal housing paradigm first mentioned by Hebb (1947), seeking to explain the effects of environment and experience on the brain and its functions in animals exposed to physical stimulation — running on a wheel, going in and out of a tunnel, etc., cognitive as exploration of the environment, manipulation of objects, etc., sensory, visual and auditory stimuli, etc., and social, coexistence and interaction with other animals (Diaz et al., 2016; Fischer, 2016; Queen et al., 2020). These elements are frequently changed to stimulate exploration through novelty (Vasquez et al., 2014; Queen et al., 2020).

Hebb (1947) found that early EE improved problem-solving ability in adult rats. Little more than a decade later, Krech et al. (1960) and Rosenzweig et al. (1962) continued their studies on EE and discovered that it produces an increase in cortical weight in the brain. Since then, there have been many investigations concerned with EE’s effects on different pathologies, especially neurological, psychiatric, and psychological, along with their comorbidities, as EE influences the neurological scope.

**Figure 2:**
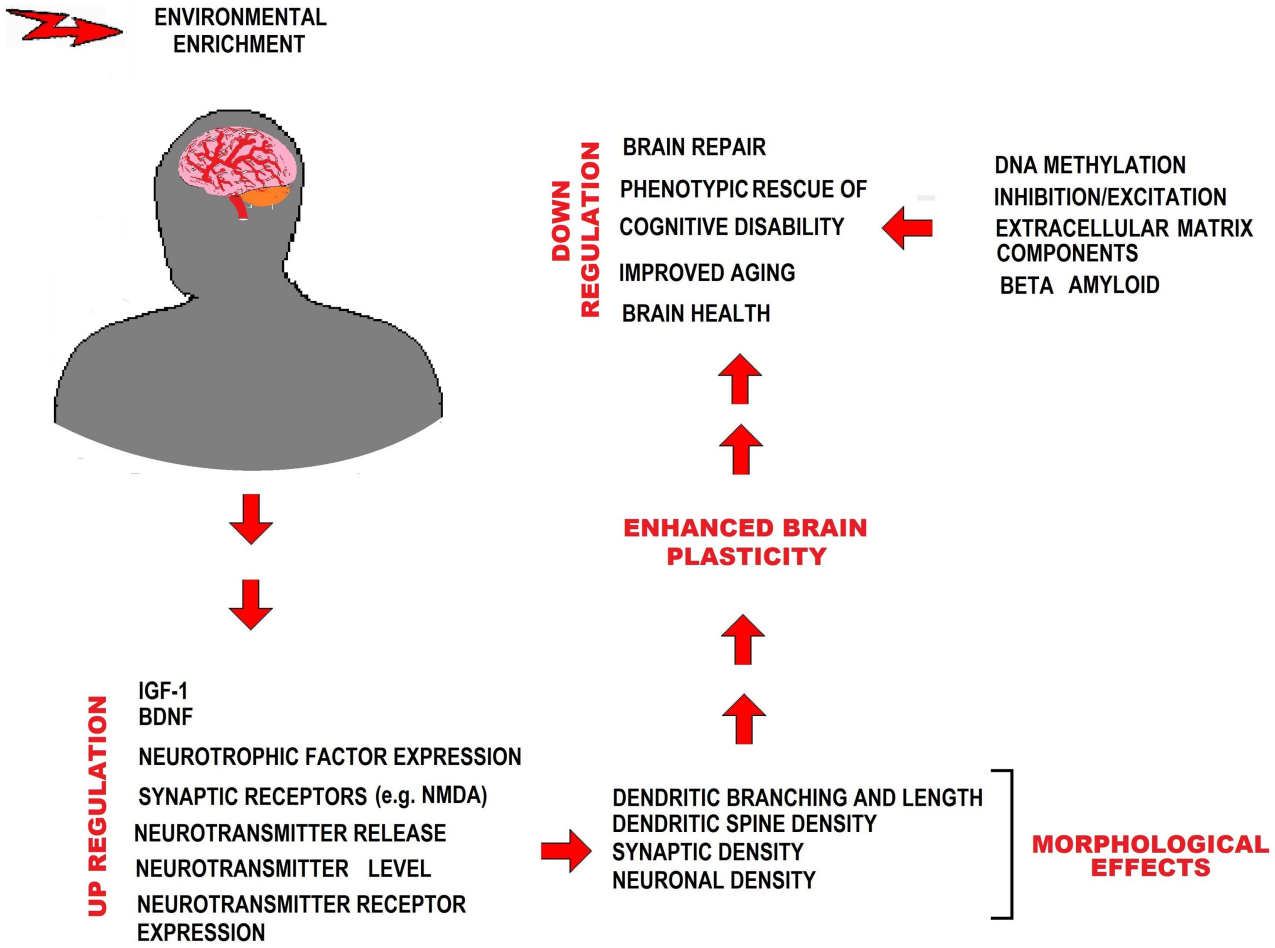
EE counteracting effects on MetS-associated CNS impairment. The figure shows different ways in which EE exerts neuroprotection from impairment by MetS. IGF-1, Insulin-like growth factor 1; BDNF, Brain-derived neurotrophic factor; NMDA, N-methyl-D-aspartate.

The neuroprotective effects of EE in the context of MetS have been associated with several factors. These include the upregulation of brain-derived neurotrophic factor (BDNF) and insulin-like growth factor-1 (IGF-1), which play crucial roles in neuronal growth, preservation, and neuroplasticity modulation, among other functions (Parks et al., 2008; Loprinzi et al., 2018; Sungkarat et al., 2018). EE has been reported to reduce oxidative stress (Rytz et al., 2020) and attenuate neuroinflammation (Queen et al., 2020), counteracting the pathophysiological conditions that contribute to developing brain degenerative disorders associated with MetS. BDNF availability influences neuronal proliferation in the hippocampus and affects synaptic plasticity, synaptic density, and neurotransmitter levels and release (Vaynman et al., 2004; Zia et al., 2021). EE has positive effects on glial cells, enhancing astrocytic volume and complexity, promoting myelination by oligodendrocytes, and facilitating microglial proliferation. These effects are essential for neural plasticity and cognitive functions (Zheng et al., 2019; Leardini-Tristão et al., 2020; Augusto-Oliveira and Verkhratsky, 2021; Verkhratsky et al., 2021). In addition, EE stimulates angiogenesis, prevents amyloid β accumulation, and induces neural progenitor cells’ proliferation (Yu et al., 2014; Gergerlioglu et al., 2016). In the brain, EE has been shown to increase hippocampus volume, in close association with improved cognitive performance (Gergerlioglu et al., 2016) (Figure 2).

Despite this paradigm being conceived for animal housing, its principles have been used in cognitive, sensory, social, and physical stimulation programs for humans, from early childhood to old age, showing that brain plasticity is present throughout the entire life cycle and not only during the first years of development (Vasquez et al., 2014). The CNS plasticity allows it to adapt, responding to the environment and its demands, remodeling neurons, making new synaptic connections, and via neurogenesis. All these events are driven by a stimuli-enriched environment and the acquisition of new skills (Vasquez et al., 2014).

Here, we briefly review brain neurodevelopmental milestones and the deleterious effects of MetS on the CNS at each life stage, and the contributions that EE models can offer to improve CNS health over the lifespan (Table 1).

**Table 1:** EE protocols used in humans and animals, including EE type, components, and duration.

Reference	Subjects	Main findings	Environmental enrichment	Duration
Environmental enrichment in childhood				
Humans				
Ortega et al. (2022)	100 participants, 8–11 years old	Exercise improved intelligence and cognitive flexibility in obese and overweight children	3–5 weekly 90-min sessions of muscle strength and high-intensity aerobic exercise	5 months
Corripio et al. (2012)	73 prepubertal obese children, 8 ± 1 years old	Compared with non-obese controls, BDNF tended to be lower in prepubertal obese children and higher after the exercise program.	Adapted exercise program	2 years
El-Alameey et al. (2019)	40 obese children, 10–15 years old	BMI and waist circumference diminished. Body fat percentage and adiposity index diminished. Systolic and diastolic blood pressure dropped. Serum cholesterol, triglycerides, LDL, glucose, BDNF, and insulin levels decreased.	30–45 min of moderate exercise 3 times/week. Participants informed their dietary history including type of food, eating behavior, and physical activity	1 year
Fang et al. (2019)	420 overweight and obese children with at least 1 MetS marker	Improved metabolic health and fewer unhealthy behaviors in obese children.	WeChat is a social media platform. A combination of individuals and teams to increase participants’ engagement. Skipping rope plus the gamification (Points, ranking, badges, punishments, and rewards)	6-month intervention and a 12-month follow-up
Latomme et al. (2022)	47 children (46.8% male), 9.69 ± 0.60 years old	Acute physical activity increased BDNF and improved some executive functions (i.e., inhibition) in children. Chronic physical activity was not associated with chages in executive functioning or BDNF levels	Maximal physical exercise test on an Ergoline 200-cycle ergometer.	1 month
Animals				
Madhavadas et al. (2017)	Male Sprague–Dawley rat pups with induced obesity	Improved insulin sensitivity, increase in total hippocampal volume, and in neuronal density in the CA1 area of the hippocampus. Improved memory.	EE groups were exposed to enriched housing conditions (81.5 × 61 × 45 cm cages) for 6 h daily. 8–10 rats were housed per cage for social stimulation. The cage contained exploratory materials like plastic tunnels, metal platforms, balls, rattle, ladders, and toys of various sizes and textures. No running wheels.	15 days
Pamidi et al. (2022)	Male Wistar pups with induced diabetes	EE substantially preserved neuronal arborizations in the hippocampus	EE cages 50x50x29 cm, fitted with various items like rotating wheels, plastic tubes, and objects of different dimensions and colors. Rats were housed in EE cages for 6 h daily.	1 month
Prabhu et al. (2022)	Male Wistar pups, with induced obesity	Mean body weight, BMI, tunica intima thickness, media layer, and percentage of collagen fibers in artery were lower in EE rats	EE cage 52×32 cm and husked bedding. Each EE cage contained tubes, running wheel, ladder, and cubes. Three rats were placed in the cage at a time and allowed to explore for 1 h daily.	3 months
Environmental enrichment in adolescence				
Humans				
Chen et al. (2016)	50 obese adolescents (age 12–15 years)	Improved executive functions, physical fitness, and obesity status	Moderate-intensity exercises were performed 4 times per week, for 40 min per session	3 months
Lee et al. (2012)	45 obese male adolescents (12–18 years)	Decrease in total and visceral fat and intrahepatic lipid were observed in both exercise groups. Improved insulin sensitivity was observed in the resistance exercise group	Aerobic exercise program 3 times/week for 60 min/session with treadmills, ellipticals, or stationary bikes. Resistance exercise included a series of 10 whole-body exercises x 3/week of 60 min/session. Each training session included leg press, leg extension, leg flexion, chest press, latissimus pull down, seated row, biceps curl, and triceps extension with stack weight equipment	3 months
Lee et al. (2014)	45 obese adolescents with type 2 diabetes, 16.4 ± 1.4 years old	Increased BDNF serum levels, decreased body weight, body fat percentage, and BMI	Aerobic exercise 3 weekly sessions, 40–60 min each.	3 months
Staiano et al. (2013)	54 obese adolescents (age 15–19 years)	Cooperative exergame players lost substantially more weight and increased self-efficacy compared with controls. Both exergame conditions increased peer support.	Play Nintendo Wii Active game (involving gross motor movements) for 30–60 min 5 days/week in cooperative or competitive groups, with each condition led by a different adult coordinator, who encouraged completion of each daily exergame routine through periodic verbal reinforcement.	7 months
Walsh et al. (2018)	202 obese adolescents (14–18 years old) with diabetes type 2 risk factors	Increase in BDNF levels and decrease in fasting glucose.	Aerobic and resistance training 4 times/week	6 months
Animals				
Díaz de León-Guerrero et al. (2022)	Male C57BL/6 N adolescent mice on a high-fat diet	EE improved glucose metabolism, increased insulin signaling in the liver, reduced hepatic steatosis and inflammation, and increased lipolysis and browning in white adipose tissue. EE reduced inflammatory signaling and increased anorexigenic signaling in the hypothalamus in high-fat-diet-fed mice.	EE housing conditions consisted of large cages (32 × 88 × 47 cm) with plastic tunnels, wood and plastic toys, cardboard boxes and glass jars. No running wheels.	3 months
Hernández-Ramírez et al. (2022)	Male 21-day-old C57BL/6 J mice exposed to high-fat-diet	Voluntary physical exercise improved glucose tolerance, halted weight gain and fat accumulation, mitigated high-fat-diet-induced spatial and recognition memory impairments.	After 12 weeks of high-fat diet intake, a running wheel was placed in the cages. Mice completed another 12 weeks, this time with voluntary running and still on the high-fat-diet.	3 months voluntary exercise
Higarza et al. (2021)	Male Sprague–Dawley rats, 4 months old, with high-fat and high-cholesterol diet	EE exposure improved cognitive functions like learning, memory, and attention in animals with MetS-like symptoms	EE cages (76.5 × 48 × 81 cm) with different stimulating objects, including platforms, tubes, little houses, running wheels, balls, and toys made of different materials, textures, shapes, sizes, and colors.	1 month
Environmental enrichment in adulthood				
Humans				
Smith et al. (2010)	124 men and women, ≥35 years old, hypertensive, sedentary, and obese or with overweight	Improvement in executive function, memory, learning, and psychomotor speed	30-min supervised aerobic exercise program 3 times per week. Weekly counseling sessions delivered in a group setting focused on teaching behavioral strategies for weight loss.	4 months
Domínguez-Sanchéz et al. (2018)	51 men (18–30 years old), physically inactive, with abdominal obesity and overweight	Increase in BDNF and neurotrophin-4/5 levels	High intensity and resistance training	3 months
Mueller et al. (2015)	16 overweight or obese men and women (age 27 ± 6 years)	Decrease in BMI and serum leptin concentrations, rise in high-density lipoprotein-cholesterol, and increase in BDNF concentrations. Increase in gray matter density in the left hippocampus, insular cortex, and left cerebellar lobule. Changes in diffusivity parameters in surrounding white matter structures and in the corpus callosum.	60 min of supervised physical training twice a week	3 months
Wing and Look AHEAD Research Group (2021)	5.145 men and women with type 2 diabetes and obesity	Fewer depressive symptoms, decreased neuropathy, and increased cerebral blood flow.	(175 min/wk. of moderate intensity activity)	10 years
Animals				
de Souza et al. (2019)	3-month-old male Swiss mice fed with a high-fat, cholesterol-enriched diet (HFECD; 20% fat and 1.5% cholesterol)	EE starting 4 weeks after the beginning of HFECD in mice, prevented HFECD-induced spatial memory and object recognition impairment, which were evaluated in T-maze and object recognition tests. EE did not counteract BDNF and IL-6 decreases in the hippocampus.	EE mice were housed in large plastic boxes (44 cm × 32 cm × 18 cm), containing a variety of stimuli: a running wheel, plastic tubing, ladders, rubber balls and a wood shelter,	1 month
Bayat et al. (2015)	8–10 weeks old Male Wistar rats with chronic cerebral hypoperfusion	The rats subjected to EE exhibited a significantly lower number of working errors and a better memory performance.	Animals in EE were housed in a large cage (90 × 75 × 45 cm, 12 animals per cage) containing toys, plastic tunnels, climbing ladders, running wheels, and stairs	10 weeks
Gergerlioglu et al. (2016)	10–12 weeks old male Wistar strain rats with high-fat or high-sucrose diet	High-fat diet consumption resulted in impaired hippocampus-dependent spatial learning and memory performance, whereas EE improved the cognitive deficits. EE increased glucocorticoid and mineralocorticoid receptor expressions.	The animals in EE group were housed in specially designed cages (90 × 75 × 45 cm) containing a variety of stimulating objects like toys, platforms, running wheels, tunnels, balls, and stairs.	1 month
Díaz de León-Guerrero et al. (2022)	6–7 weeks old C57BL/6 N male mice on a high-fat diet	EE improved glucose metabolism, increased insulin signaling in the liver, reduced hepatic steatosis and inflammation, and increased lipolysis and browning in the white adipose tissue of high-fat diet-fed mice. EE reduced inflammatory signaling and increased anorexigenic signaling in the hypothalamus of high-fat-diet-fed mice	EE housing conditions were provided by large cages (32 × 88 × 47 cm) with plastic tunnels, wood and plastic toys, cardboard boxes, and glass jars.	3 months
Brenes et al. (2016)	Juvenile male Wistar rats	Social and physical EE have differential effects on brain plasticity, cognition, and ultrasonic communication. Physical EE promoted neurogenesis in the dentate gyrus of the hippocampal formation, but not in the subventricular zone, and affected microRNA expression levels, upregulating activity-dependent miR-124 and miR132. Concomitant improvement in cognition were observed. In contrast, social EE had only minor effects on brain plasticity and cognition, but led to increased prosocial 50-kHz USV emission rates and enhanced social approach behavior.	EE protocol without running wheels was used. EE consisted of a big cage (380x200x 590 mm) containing the following items: bedding, pieces of towel paper, roll papers, grass nests, wood sticks, feeding balls filled with towel paper and food pellets, cardboard boxes, and nonchewable plastic, glass and metal objects. There were groups of 2 rats (physical EE) and of 6 rats (social EE)	1 month
Leardini-Tristão et al. (2020)	12-week-old male Wistar rats with cerebral hypoperfusion	Early moderate physical exercise normalized functional capillary density and reduced leukocyte rolling in the brain of rats with chronic cerebral hypoperfusion. These effects were accompanied by restored synaptic protein and improved cognitive function. Early moderate exercise improved astrocytes coverage in blood vessels of the cerebral cortex and hippocampus, decreased microglial activation in the hippocampus, and improved structural capillaries in the hippocampus.	All the animals were adapted to a treadmill for rats. Physical exercise consisted of 30-min sessions, 3 times a week for 12 weeks at 60% of the maximal speed	3 months
Zheng et al. (2019)	From birth to 4 months’ old male C57BL/6 mice	Mice EE-reared had better acquisition and consolidation of memory. They showed reduced anxiety in novel environments, enhanced social interactions, and improved motor ability, and learning.	EE was provided by 50x36x28 cm cages containing objects of various shapes and textures (small plastic houses, tunnels, plastic blocks) and additional bedding materials (shredded paper, paper roll, cotton wool and textile pieces), a stainless steel spice cube, containing various species, and running wheels.	4 months
Environmental enrichment in old age				
Humans				
Hamer et al. (2018)	10-year follow-up of 10.652 (65 ± 10 years old) men and women from the English Longitudinal Study of Ageing	Physically inactive women experienced a greater decline in memory and in executive function ability compared with the vigorously active reference group. In men, there were no differences in memory but small differences in executive function between inactive and vigorously active ones were observed.	Self-reported physical activity was measured using 3 questions on the frequency of participation JECH Online Research report in vigorous, moderate and mild intensity physical activities (more than once per week, once per week, 1–3 times per month, hardly ever).	10 years
Sungkarat et al. (2018)	66 older adults (mean age = 67.9 years)	Plasma BDNF level increased in the Tai Chi group. Tai Chi training improved memory and the mental switching component of executive function.	The participants learned Tai Chi with a certified instructor and then practiced at home 50 min/session, 3 times/wk	6 months
Niemann et al. (2014)	92 older adults, 62–79 years old	Cardiovascular and coordination training led to increased hippocampal volume	Training interventions 3 times per week, 45–60 min each. They included coordination training, cardiovascular training, stretching, and relaxation training	12 months
Rytz et al. (2020)	236 men and women 55–80 years old, sedentary, with MetS	Reduced oxidative stress	6-months of aerobic exercise intervention	6 months
Ten Brinke et al. (2018)	124 men and women 65–85 years old	Improvement in executive functions, specifically response inhibition, probably associated with a decreased correlation between the left dorsolateral prefrontal cortex and bilateral medial temporal gyrus regions.	Computerized cognitive training and brisk walking, 1 h class x 3 times/ week plus 3 times x/week home-based training	2 months’ intervention, 1 year follow-up
Animals				
McMurphy et al. (2018)	10-month-old female C57Bl/6 mice	Short-term EE activated the hypothalamic-sympathoneural-adipocyte axis in 10-month-old mice. Long-term EE reduced adiposity, improved glucose tolerance, decreased leptin levels, enhanced motor abilities, and inhibited anxiety. EE decreased age-related liver steatosis, reduced hepatic glucose production, and increased hepatic glucose uptake and adipose tissue, improving glycemic control. EE-induced liver modulation was associated with a suppression of protein kinase Cε. EE down-regulated the expression of inflammatory genes in the brain, adipose tissue, and liver.	EE was provided by large cages (63 cm x 49 cm x 44 cm, 5 mice per cage) supplemented with running wheels, tunnels, igloos, huts, retreats, wood toys, a maze, and nesting material	Short term: 6 weeks. Long term: 12 months
Huang et al. (2020)	Male 5- or 3-week-old C57BL/6 mice, with excess adiposity	EE upregulated adipose phosphatase and tensin homolog deleted on chromosome ten (PTEN) expression, increased hormone-sensitive lipase phosphorylation, and reduced adiposity. PTEN regulation was controlled by the hypothalamic-sympathoneural-adipocyte axis. Hypothalamic BDNF was the upstream mediator, dependent on sympathetic innervation.	Mice were housed in large EE cages (20x90x76 cm) supplemented with running wheels (1 wheel in midsize cages; 2–3 wheels in large cages), maze tunnels, huts, retreats, wood logs, and nesting material.	5-week-old:1 month. 3-week-old: 4 months
Kempermann et al. (2002)	10 months’ old female C57BL/6 mice	Adult hippocampal neurogenesis in mice living in an EE was 5-fold higher than in controls. Newly generated astrocytes were observed. Improvement in learning parameters, exploratory behavior, and locomotor activity. EE-mice had a decreased lipofuscin load in the dentate gyrus, indicating decreased nonspecific age-dependent degeneration.	EE consisted of a large cage with approximately 1 m^2^ floor area. This cage was equipped with a rearrangeable set of plastic tubes, a small running wheel, nesting material, and toys.	10 months

## Childhood

### CNS development in childhood

The biological, psychological, and emotional changes that take place between birth and the end of puberty are referred to as childhood development. To ensure that developmental trajectories are on track and evolution in each area is properly monitored, milestones have been established. Aside from that, anthropometric markers commonly follow recognized growth curves. Children’s growth, gross and fine motor, social, emotional, verbal, and cognitive developments are monitored at every physician visit (Ronan et al., 2021).

Central nervous system development begins prenatally, and certain regions and neural circuits go on developing postnatally until the mid-20s or even 30s (Gogtay et al., 2004; Petanjek et al., 2011). The fetal period comprises growth, differentiation, and organogenesis, and brain weight increases about 40-fold (Müller and O’Rahilly, 2006). CNS development involves changes regarding cellular compartments, cell types, and synaptic circuits. These changes follow in response to gene–environment interactions during prenatal months and to postnatal experiences-genetic interactions.

During the first three weeks after fertilization, gastrulation sets up the three germ layers, and the neural plate is transformed into a neural tube by primary neurogenesis. Cell line transformation taking place during gastrulation sets the basis for all subsequent embryonic development (Stiles and Jernigan, 2010; Nikolopoulou et al., 2017). The anterior portion of the tube becomes the forebrain, including the cerebral hemispheres, diencephalon — thalamus and hypothalamus — and the basal ganglia. The middle portion of the neural tube gives rise to the midbrain, connecting the diencephalon to the hindbrain, and the rear-most portion yields the hindbrain. The remaining cells give rise to the spinal cord. Once the general structure of the neural tube has been laid out, the cells in the inner part of the tube — the ventricular zone — show a logarithmic growth (Tierney and Nelson, 2009).

The cerebral cortex comprises six layers of tissue several millimeters thick in an inside-out pattern of migration. About 80% of migrating neurons send signals to different layers of the cortex and other parts of the brain. Glial nonneuronal brain cells are involved in producing myelin or removing debris. After the cells are born, they move beginning from the ventricular zone to the outside zone of the developing brain. The completion of the six layers takes 25 weeks after conception. Interneurons follow a pattern of tangential migration and make communications with pyramidal cells of the cortex (Tierney and Nelson, 2009). Following the migration, cells differentiate into mature neurons with axons and dendrites (Oppenheim and Johnson, 2003). Axons grow through growth cones at the ends of the axons. Dendrites grow in a process that is thought to be driven by genes controlling calcium-regulated transcription factors (Aizawa et al., 2004). Neurons connect to one another by synaptic connections essential for brain function. The first synapses occur around the 23rd week of gestation (Molliver et al., 1973), but the peak is not before the first year of life. Neuron production and synapses proceed massively first and then gradually decrease. Different structures of the brain reach their peak of synapse production at different points, affecting the plasticity timing of each region. The later the peak synapse production, the longer the region remains plastic (Tierney and Nelson, 2009).

Brain developmental stages are mainly gene driven before synaptogenesis, while pruning is largely experience-driven. Pruning of areas involved in higher cognitive functions like inhibitory control and affective modulation keeps on through adolescence (Huttenlocher and Dabholkar, 1997). These adaptive changes from synapses’ overproduction to reduction are crucial for the developing mind, allowing neuronal networks responsible for behavioral development to be fine-tuned and altered as needed (Tierney and Nelson, 2009).

Myelination is the last process of brain development. Fatty cells wrap neurons, facilitating neuronal activity and communication. Myelinated axons (isolated) transmit electrical signals faster than unmyelinated axons. Myelination timing varies across the different brain areas. It is earlier in motor areas, concurrent with the preschool period, while in areas involved in cognitive abilities, it is not observed but until adolescence or even early adulthood (Tierney and Nelson, 2009).

At birth, CNS anatomy resembles adult one, the neocortex has neurons established, then, neurogenesis continues in the cerebellum and the generation of glial cells throughout infancy and early childhood (Pakkenberg and Gundersen, 1995; Pakkenberg et al., 2003). An outgrowth of dendrites and axons, followed by synaptogenesis and myelination characterizes early postnatal development. Later, in the three postnatal years, the growth of the brain continues to reach its greatest weight (Dekaban, 1978). Neural circuits structural changes, and molecular reorganization continue through late childhood and adolescence at the same time getting higher-order cognition and complex behavior (Silbereis et al., 2016).

The growing brain is particularly susceptible to environmental stimuli, from *in-utero* life through the first few years of life (Miguel et al., 2019). Experiences that occur during this time along with epigenetic modifications, modify brain’s structure and function permanently, and subsequently affect a person susceptibility to mental and neurologist diseases. Maternal impact on fetal growth includes the mother’s age, socioeconomic level, maternal health, usage of drugs, and nutrition (Miguel et al., 2019).

Developmental delays and an increased risk of many different mental health issues are linked to poor intrauterine growth. Inattention, hyperactivity, and internalizing issues in infancy and adolescence were linked to extremely low birth weight, according to one meta-analysis results (Mathewson et al., 2017). All these issues eventually lead to greater social problems, depression, and anxiety in adulthood.

From conception through childhood, the intestinal microbiome’s makeup has an impact on a person’s health, and dysbiosis has been linked to several disorders. It is known that it is constantly evolving, and several factors influence its composition (Ronan et al., 2021). Any deviation from expected developmental milestones might be a sign of the onset of an illness. It is vital to look into how the gut microbiome develops and how the microbiota interacts with other organs. The gut microbiota, consisted of all the community of living microorganisms residing in it, should be considered an organ system that significantly impacts diseases in children and adults, including autism spectrum disorders (ASD), attention deficit hyperactivity disorder (ADHD), asthma, and allergies (Ronan et al., 2021).

Bacteria represent nearly 90% of all taxonomic microbiota (Thursby and Juge, 2017). Gut dysbiosis has been implicated in the development of different chronic diseases like metabolic disorders, including metabolic syndrome, type 2 diabetes (D2M), and obesity (Holmes et al., 2011; Carding et al., 2015; Zhang et al., 2015; Baothman et al., 2016; Org et al., 2017). Gut bacteria produce and release bioactive compounds with neurotransmitter potential, and the gut-brain axis has long been described. The gut microbiome produces serotonin, dopamine, noradrenaline, acetylcholine, and GABA (Galland, 2014; Strandwitz, 2018; Del Colle et al., 2020). Intrinsic host factors as genetics and gut microbiome, and extrinsic factors like diet and lifestyle, eventually interact and determine MetS development (Hills et al., 2019; Gildner, 2020). Obese people’s microbiomes may be more prone to get energy from food, increasing body mass index (BMI) and insulin resistance (IR), both conditions for MetS (Mazidi et al., 2016).

A noteworthy theory proposed that individuals born small for gestational age that experienced rapid weight gain faced an elevated risk of chronic diseases in the long term (Barker et al., 1993). These risks included insulin resistance (Hales et al., 1991), D2M (Barker et al., 1993), systolic hypertension (Barker, 1992), and coronary heart disease (Barker and Martyn, 1992), among others, all associated with MetS. In line with these ideas, the Forsdahl-Barker hypothesis proposed that MetS, also known as syndrome X, could be regarded as the small baby syndrome based on its fetal origin (Brenseke et al., 2013).

These observations suggest that fetal programming and epigenetic factors are associated with susceptibility to chronic disease later in life. Low birth weight infants have high levels of adiposity and an increased vulnerability to developing chronic diseases (Hales and Barker, 2001). Barker et al. (1993) proposed a relationship between intrauterine malnutrition, insulin resistance, impaired glucose tolerance, and MetS development in adulthood (Jebasingh and Nihal, 2022). Maternal malnutrition, excessive maternal weight gain during pregnancy, and maternal smoking can contribute to increased metabolic risks in newborns (Rogers, 2019). Therefore, interventions should focus on promoting healthy maternal nutrition during pregnancy, encouraging breastfeeding, avoiding rapid weight gain during infancy and prepubertal stages, and promoting healthy lifestyle habits (Gregory, 2019).

### Effects of MetS in CNS in childhood

MetS is a cluster of cardio-metabolic risk factors associated with an increased risk of cardiovascular disease in children and adults. However, there is not yet a straightforward, universally recognized definition of MetS in children to ensure high sensitivity and stability of diagnosis. At least 40 definitions of MetS in children have been established (Reaven and Hoffman, 1987). Several consensus statements and national guidelines fully agree that the main features defining MetS in children and adolescents include (1) disturbed glucose metabolism, (2) arterial hypertension, (3) dyslipidemia, and (4) abdominal obesity (Weihe and Weihrauch-Blüher, 2019). Yet, cut-off values are heterogeneous in this age range (Tropeano et al., 2021; Hampl et al., 2023).

Insulin sensitivity, serum lipid concentrations, and anthropometrical variables change with age, making a pediatric definition of MetS difficult. The International Diabetes Definition (IDF) (Ford and Li, 2008) states that children over 10 years can be diagnosed with MetS, but children under 10 who meet the criteria should be considered a risk group. This definition is more precise and user-friendly in clinical practice than the other ones. It has the undeniable advantage of requiring readily available measurements without using many reference tables and measurements (Tropeano et al., 2021). The IDF definition is primarily used in Europe to diagnose MetS in children and adolescents (Zimmet et al., 2007), and is used in this review.

In 2009, the consensus statement from the American Heart Association emphasized the need for additional studies on children and adolescents to settle if MetS risk factors might group together and predict future illness. (Steinberger et al., 2009). Based on Magnussen et al. investigation, the most important finding was that high BMI predicts MetS as well as, or better than the other clinical features included in MetS definitions considered in the study, which were subclinical atherosclerosis, carotid intima-media thickness, and T2D (Magnussen et al., 2010). More studies should aim to arrive at a consensus and predict the risk of MetS in adulthood.

The WHO defines overweight and obesity as abnormal or excessive fat accumulation that may impair health. The body mass index (BMI) is routinely used to categorize adulthood overweight and obesity. For children under 5 years, a BMI higher than 2 or 3 standard deviations above the WHO Child Growth Standards median determines overweight or obesity, respectively (World Health Organization, 2021). BMI measurements alone provide an easy and informative measure of subsequent cardiometabolic risk (Koskinen et al., 2017).

Obesity and overweight are alarmingly growing global health problems (Tiwari and Balasundaram, 2023). Between 1975 and 2016, there was an eight-fold increase in the prevalence of obesity in those between the ages of 5 to 19. Industrialized nations have higher rates of childhood obesity, and this problem is becoming widespread (Balasundaram and Krishna, 2023). Obesity predisposes to insulin resistance-type II diabetes due to the concurrent malfunction of adipocytes, myocytes, and hepatocytes, oxidative stress, and inflammation (Morrison et al., 2007, 2008; Tilg and Moschen, 2008; Shulman, 2014). One study found alterations in lipid and fatty acids metabolism and inflammatory response in obese children compared with their normal weight counterparts, analyzing the transcriptomic profile of adipose tissue from the periumbilical area. They highlighted that obesity may trigger inflammation and neuroinflammation from a pediatric age (Zingale et al., 2022). Because of its prevalence and importance, obesity is the central and more studied feature in the investigations about MetS in childhood.

Hypertension is less frequent in children than in adults. Still, as in adults, obesity is intrinsically linked to hypertension and prehypertension in children and adolescents (Litwin and Kułaga, 2021). Historically, secondary hypertension is the most common form of childhood hypertension. However, the situation has changed in the last 20 years, and primary hypertension is now the leading cause of hypertension in children over 6 years, and in adolescents, in particular (Gupta-Malhotra et al., 2015).

Inflammation and microvascular damage in MetS increase susceptibility to brain damage and cognitive impairment (Yates et al., 2012). The CNS may also be negatively affected by obesity, as shown by their association with cognitive decline and an increase in brain injury vulnerability (In a recent publication, a group of investigators conducted a systematic review, and a meta-analysis to quantify and summarize evidence on the association between ADHD) and T2D (Garcia-Argibay et al., 2023a). The meta-analysis showed that individuals with ADHD had more than a twofold risk of T2D, and the main drivers of such association were substance use disorder, depression, and anxiety, while cardiovascular and familiar risks played a smaller role (Garcia-Argibay et al., 2023b). ADHD has an estimated global prevalence of 5–10% in children, and 2–5% in adults, and it is a heterogeneous neurodevelopmental condition defined by impairment levels of inattention, hyperactivity/impulsivity, or both (Garcia-Argibay et al., 2022).

Besides, children with MetS may present alterations in their brain structure and/or function. Several groups of investigators analyzed the relationship between cognition and some important MetS components. Mamrot and Hanć (2019) reviewed the connections between obesity and executive function, a cognitive domain dependent on the frontal lobe, in children and adolescents. The investigators found a significant association between executive dysfunction and excess BMI in the children studied. In 2020, another analyzed BMI and its association with the thickness of the cerebral cortex in 3190 children, founding that greater BMI was associated with a decreased cortical thickness (Laurent et al., 2020).

Scudder et al. (2015) investigated the association between MetS risk factors and cognitive control in preadolescent children and found better inhibitory control and increased cognitive flexibility in children without risk factors for MetS compared to at-risk children. Also, the result of a systematic review and meta-analysis of the incidence of depression and anxiety symptoms between overweight/obese or non-overweight/non-obese in Chinese children and adolescents showed a higher prevalence in the overweight/obese group. Physical exercise and psychological interventions were then recommended to prevent the associated psychological problems (Wang et al., 2019a).

Despite the difficulty in setting cut-off values for MetS components in the pediatric population (Weihe and Weihrauch-Blüher, 2019), abdominal obesity is one of the key features defining MetS in children and adolescents (Weihe and Weihrauch-Blüher, 2019). The brain-derived neurotrophic factor (BDNF), a crucial regulator of neuronal growth, survival, and neuronal preservation (Tapia-Arancibia et al., 2004), plays a major role in feeding behavior, food intake regulation, energy metabolism, and body weight control (Skledar et al., 2012; Bathina and Das, 2015). However, the relationship between BDNF and obesity in children is not clear, and the reported findings are inconclusive (Corripio et al., 2012; Roth et al., 2013). Rozanska et al. (2020) reported lower BDNF levels, while Boyuk et al. found higher BDNF blood levels, and Lee et al. (2014), studying adolescents, reported similar resting BDNF levels in D2M patients compared with non-diabetic counterparts (Boyuk et al., 2014). BDNF was negatively associated with age (Suwa et al., 2006).

Childhood obesity is associated with many cardiovascular risk factors related to endothelial dysfunction, and cardiovascular morbidity and mortality. Obstructive sleep apnea (OSA) is usually caused by obesity and is another factor linked to cardiovascular alterations with cognitive complications (Konstantinopoulou and Tapia, 2016; Berger and Polotsky, 2018). OSA-obesity patients have recurrent hypoxemia episodes, which can provoke increased sympathetic activity, oxidative stress, and inflammation, contributing to endothelial dysfunction (Dempsey et al., 2010).

Given the obesity-OSA relationship, and its increasing incidence, these two conditions can contribute to endothelial dysfunction and cardiovascular morbidity (Katz and Pillar, 2016). Makhout et al. found an interaction between OSA, endothelial function, and BDNF levels in obese children, while BDNF levels were not different in obese children with or without OSA (Makhout et al., 2022).

### EE counteracting effects on MetS-associated CNS impairment in childhood

EE improves memory and mitigates learning deficits in brain injury models (Rojas-Carvajal et al., 2022). In rodents, exposure of offspring with induced obesity or diabetes to EE, incorporating physical exercise through the use of running wheels, resulted in noteworthy preservation of neuronal arborizations in the hippocampus. This intervention also led to decreases in body weight, collagen fiber deposition in artery walls, and tunica intima thickness (Pamidi et al., 2022; Prabhu et al., 2022). In rat models that did not include exercise, EE improved insulin sensitivity, increased hippocampal volume, augmented neuronal density, and enhanced memory function in pups with induced obesity (Madhavadas et al., 2017). Healthy life habits like increasing physical activity and reducing caloric intake on a daily basis help to reduce body weight and cardiovascular and metabolic disorders risk all over the lifespan.

After 1 year of a lifestyle intervention program in children, a team of researchers created a study to assess the association between serum BDNF levels and MetS components. Primary lifestyle intervention consisted of changes in dietary habits and physical activity. Regarding exercise, it was a modified routine of three times per week, consisting of 30 to 45 min of moderate exercise. They found lower concentrations of BDNF in obese compared with control children. The study results revealed that BDNF may be a key player in the pathophysiology of childhood obesity and can help identify children at risk for cardiovascular and metabolic problems (El-Alameey et al., 2019).

In 2019, Fang et al. conducted a study arguing that traditional or supervised exercise interventions were excellent at enhancing metabolic health. However, it had a lack of long-term impact and low involvement. Instead, they thought gamification could increase engagement and achieve sustained exercise intervention effects. Then, they proposed that mobile technologies like WeChat could provide low-cost scale platforms. Based on these suppositions, the investigators proposed a novel exercise intervention named the S&G exercise, which combines supervision with gamification to social incentives to improve metabolic health among overweight and obese children. In their study, 420 overweight and obese children with at least one marker of MetS were analyzed in an exercise intervention package, which included interventions combining integrated social incentives with gamification theory. According to their results, the study could explore a low-cost, easy-to-popularize, and effective exercise intervention model for improving metabolic health among the obese. In their opinion, investigators sustained that the S&G exercise intervention package could predict an innovative exercise intervention to develop improvements in metabolic health and in unhealthy behaviors among obese children (Fang et al., 2019).

Current environmental and lifestyle factors reduce the possibilities for energy expenditure through active play to obesity children. In addition, the availability of high-energy-dense foods and beverages has increased. The evidence shows that many children’s sedentary habits are rising, and simultaneously, physical activity seems to be dropping. These two facts combined are likely linked to obesity and put danger to children’s health. Environmental enrichment (EE) and its components, like regular physical exercise, may be fruitful in analyzing aspects of children’s risk of obesity and its consequences.

The first case–control study with a prospective 2-year follow-up was conducted at a pediatric endocrine outpatient center, in which the relationship between plasma BDNF concentrations and the elements of MetS in prepubertal obese children was analyzed before and after a lifestyle intervention program. As a result, prepubescent obese kids typically had lower plasma BDNF concentrations than thin peers. In youngsters who were obese, diet and exercise could enhance BDNF levels. Further studies to elucidate the exact role of BDNF in the physiopathology of obesity in prepubertal children were also recommended (Corripio et al., 2012).

A group of investigators surveyed the literature on the effects of physical training and exercise on BDNF levels in kids and teens (de Menezes-Junior et al., 2022). Inclusion criteria were any initial research that investigated the impact of exercise and physical training on plasma and serum BDNF concentrations in kids and teens. This meta-analysis found that, as compared to inactive people, adolescent athletes tended to have lower serum but higher plasma BDNF concentrations. Furthermore, among sedentary teenagers, exercise appeared to raise serum BDNF concentrations mildly (de Menezes-Junior et al., 2022).

Another study examined whether children who engaged in the short-term and long-term exercise had a better executive function and concluded that acute exercise might render some increase in BDNF and some improvement in children, but sustained exercise and BDNF were not associated with executive functions (Latomme et al., 2022).

In view of the research results commented here, pursuing on this topic investigation seems essential to reach consensus on the role and consequences of BDNF levels in obese children with or without physical activity programs.

The ActiveBrain, is another interesting program, consistent on a social platform for active and healthy ageing. The ActiveBrain project is an initiative from the University of Granada in Spain (Cadenas-Sánchez et al., 2016) born based on the certainty that children physically fit have an increase in the amount of grey matter in the calcarine cortex and frontal and temporal areas of the brain. These brain areas are crucial for learning, motor skills, visual processing, and executive function. To find out if the brains of physically fit youngsters differed from those of their less fit classmates and if this had an impact on their academic performance, the researchers compared the brains of the two groups of kids. The study design included a 20-week intervention consisting of three to five 90-min sessions each week, focusing primarily on high-intensity aerobic exercise and incorporating muscle-strengthening activities. Yet, researchers found no connection between the amount of grey matter in any brain section and muscular strength. Finally, they stressed the importance of undertaking more thorough randomized controlled trials to evaluate the impact of exercise on brain anatomy and functions, cognition, and physical and mental health. They also mentioned the necessity for greater research on the evidence and specificity of the human brain (Cadenas-Sánchez et al., 2016).

Another study was conducted to examine the association between cognitive control and the risk factors for MetS in preadolescent children while accounting for aerobic capacity and BMI. The results were in agreement with the preceding literature, as they concluded that aerobic fitness positively affected cognitive control. They showed that children without MetS risk factors had better inhibitory control and greater cognitive flexibility than those at risk. These risk factors and aerobic fitness may be crucial biomarkers of the possible cognitive effects of MetS risk in younger generations (Scudder et al., 2015).

**Figure 3:**
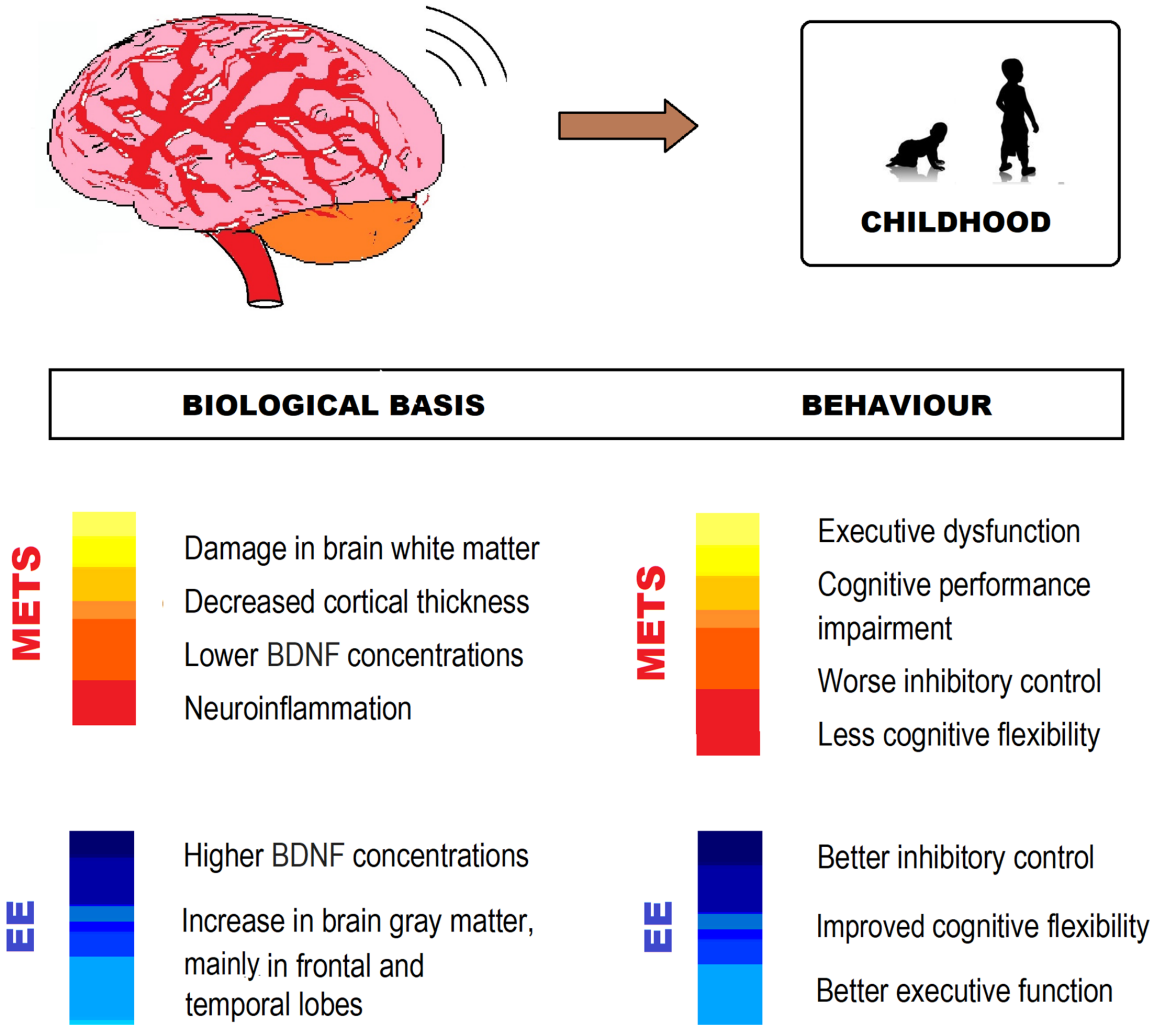
MetS-associated CNS impairment and behavioral changes, and EE counteracting effects in childhood. The figure shows different ways in which EE exerts neuroprotection from impairment by MetS. BDNF, Brain-derived neurotrophic factor.

Taking into account this evidence, it is undeniable that EE and its components, particularly physical exercise, have important neuroprotective effects and improve the health of children who already suffer from MetS or have MetS risk factors (Figure 3).

## Adolescence

### CNS development in adolescence

Adolescence is a critical period of development characterized by rapid and dynamic changes in the brain that can have long-lasting effects on cognition and behavior. The neural changes that occur during adolescence are driven by a combination of genetic and environmental factors, including hormonal fluctuations, changes in synaptic plasticity, and experiences during this period of life.

Neuroanatomically, the adolescent brain undergoes significant changes, particularly in the prefrontal cortex, hippocampus, and amygdale (Arain et al., 2013; Silbereis et al., 2016). These changes are influenced by both genetic and environmental factors, so the interplay between these has crucial implications for developmental outcomes and mental health. The prefrontal cortex, which is responsible for decision-making, planning, and impulse control, undergoes a pruning process during adolescence that reduces the number of synaptic connections, making the brain more efficient. The gray matter volume in the prefrontal cortex peaks at around 12 years of age in females and 14 years of age in males and then begins to decrease, which may account for the increased risk-taking behavior in adolescence. The corpus callosum, which connects the two hemispheres of the brain, also undergoes significant changes in adolescence, becoming thicker and more efficient in transmitting information (Arain et al., 2013; Silbereis et al., 2016).

Structural MRI studies have shown that gray matter volume in the prefrontal cortex increases throughout adolescence, peaking around 16–18 years of age, and then gradually decreasing until adulthood (Miguel-Hidalgo, 2013; Sung et al., 2021). Along with increased volume, the prefrontal cortex also undergoes changes in connectivity and myelination, which contribute to its maturation (Caballero et al., 2016).

Furthermore, adolescence is also a critical period for myelination in white matter tracts that connect different brain regions. Diffusion tensor imaging (DTI) studies have shown that white matter integrity increases during adolescence, with some tracts showing a nonlinear trajectory of development (Caballero et al., 2016). However, development is not uniform across all tracts and some of them show earlier maturation compared with others. Structural changes in the brain during adolescence include sustained myelination and pruning of connections, with gray matter volume, peaking in early adolescence and white matter volume, increasing throughout adolescence and into early adulthood. These changes are thought to reflect the ongoing refinement of neural circuits that underlie complex cognitive processes (Asato et al., 2010; Arain et al., 2013; Caballero et al., 2016).

The limbic system, which includes the amygdala and hippocampus, also undergoes significant changes during adolescence. The amygdala undergoes structural changes during adolescence, including increased volume and greater connectivity with other regions of the brain. These changes may contribute to increased emotional reactivity during this period (Scherf et al., 2013).

Metabolic changes in the brain during adolescence include changes in glucose utilization and the expression of genes related to energy metabolism. These changes may reflect the need for increased energy demands during this period of life, as well as the ongoing development of neural circuits (Oyarzábal et al., 2021).

There is growing evidence that the adolescent brain is particularly sensitive to environmental factors like stress, substance use, and nutrition (Cacciatore et al., 2022; Winters and Ingwalson, 2022). For example, exposure to chronic stress during adolescence has been associated with impaired brain development, including changes in the hippocampus, prefrontal cortex, and amygdale (Tottenham and Galván, 2016). Similarly, substance use during adolescence can have long-lasting effects on brain development as shown by reports of reduced gray matter volume in areas associated with reward processing and decision-making (Tottenham and Galván, 2016).

In addition to these structural and metabolic changes, adolescence is also a period of important cognitive and social development. This includes the development of executive functions such as working memory, inhibition, and cognitive flexibility, as well as the development of social cognition and emotional regulation.

### Effects of MetS in CNS in adolescence

The development of MetS during adolescence is influenced by a complex interplay between genetic and environmental factors. Genetic factors play a role in determining an individual’s susceptibility to MetS, and several genetic variants have been associated with the development of MetS in adolescents (Wong et al., 2022). Environmental factors, such as poor diet, lack of physical activity, and psychosocial stress, also contribute to the development of MetS during adolescence (Wong et al., 2022). Besides, studies have shown that a high intake of sugar-sweetened beverages and a low intake of fruits and vegetables are associated with the development of MetS in adolescents (Malik and Hu, 2019; Button et al., 2022). In addition, physical inactivity and sedentary behavior have been linked to the development of MetS during adolescence (De Oliveira and Guedes, 2016).

Psychosocial stress is another environmental factor that has been implicated in the development of MetS during adolescence. Stressful life events, such as parental divorce, financial strain, and academic pressure, have been associated with an increased risk of MetS in adolescents (Reid et al., 2020). Chronic stress can lead to dysregulation of the HPA axis and the sympathetic nervous system, which can contribute to the development of MetS (Stephens and Wand, 2012).

Moreover, certain environmental exposures, such as exposure to endocrine disruptors, can also influence the development of MetS. For example, exposure to bisphenol A (BPA), a chemical found in some plastics, has been associated with an increased risk of MetS in adolescents. BPA can disrupt hormone signaling pathways, leading to alterations in glucose and lipid metabolism, which are components of MetS (Trasande et al., 2012; Wu et al., 2020; Abulehia et al., 2022).

Numerous studies have investigated the interplay between genetic and environmental factors that contribute to the development of MetS during adolescence. Concerning genetics, compelling evidence indicates that specific gene variants, including FTO, MC4R, and PPARG, are linked to an elevated risk of MetS in adolescents (Szkup et al., 2018). These genes are involved in regulating critical processes such as energy metabolism, appetite control, and insulin resistance, which are all components of MetS. A study revealed that specific genetic polymorphisms were linked to MetS components, including systolic blood pressure and high-density lipoprotein cholesterol among adolescents (Kapil et al., 2018; Wong et al., 2022). Furthermore, a study found that polymorphisms in the ADIPOQ gene were associated with MetS risk in Chinese adolescents (Gao et al., 2013). Additionally, variations in the FTO gene were significantly associated with MetS and its components adolescents (Liu et al., 2013). These findings suggest that genetic testing may be beneficial in identifying adolescents at risk of MetS.

Moreover, changes in neuroanatomy and brain function during adolescence could also contribute to the development of MetS. For instance, the prefrontal cortex’s functional connectivity is crucial for executive functions, and it continues to develop during adolescence (Liu et al., 2013). Arain et al. (2013) and Scherf et al. (2013) reported that the amygdala volume increases during adolescence, and this brain region plays a critical role in emotional reactivity. These studies indicate that changes in brain structure and function during adolescence may play a role in the development of MetS.

However, it is essential to consider environmental factors in the development of MetS in adolescence. A recent study revealed that an unhealthy diet, physical inactivity, and poor sleep quality were significantly associated with MetS components in adolescents (Fatima et al., 2016; Gohil and Hannon, 2018). Another study by Wang et al. (2019b) reported that sedentary behavior and low physical activity levels were associated with higher risks of MetS and its components in Spanish adolescents. A study conducted by Wang et al. (2019a) found that low levels of vitamin D were significantly associated with the development of MetS in iranian adolescents. In contrast, a study by de Oliveira and Guedes (2018) revealed that increased physical activity was associated with lower MetS risk in Brazilian adolescents.

Several studies have explored the contribution of environmental factors, such as diet and physical activity, in the development of MetS during adolescence. a study reported a positive association between a Western diet, characterized by high intake of fat and sugar, and increased risk of MetS in adolescents (Naja et al., 2015). Similarly, De Oliveira and Guedes (2016) found that low levels of physical activity were linked to a higher risk of MetS in adolescents. These findings underscore the importance of lifestyle modifications, such as dietary changes and regular physical activity, in the prevention and management of MetS during adolescence.

Furthermore, the interplay between genetic and environmental factors in the development of MetS is critical. De Oliveira and Guedes (2016) found that the genetic risk for MetS was more pronounced in adolescents who had a high intake of fat and sugar. In another study, Aasdahl et al. (2021) showed that the interaction between a genetic variant and low physical activity was associated with an increased risk of MetS in adolescents. These findings highlight the importance of personalized interventions for adolescents at high risk of developing MetS, taking into account their genetic and environmental factors.

It is evident from these studies that both genetic and environmental factors are crucial in the development of MetS during adolescence. Further research is needed to explore the mechanisms underlying these interactions and to identify novel therapeutic targets for preventing and treating MetS during this critical period of development. With the increasing prevalence of MetS in adolescents, it is imperative that effective prevention and management strategies are implemented to mitigate the deleterious effects of this disorder.

Research has shown that alterations in the prefrontal cortex, amygdala, hippocampus, and striatum during adolescence may contribute to the development of mental and neurological disorders later in life. Reduced gray matter volume in the prefrontal cortex has been associated with an increased risk of developing depression and anxiety disorders (Asato et al., 2010; Arain et al., 2013; Miguel-Hidalgo, 2013; Scherf et al., 2013; Sung et al., 2021). Similarly, alterations in the amygdala have been linked to the development of mood disorders and anxiety disorders, while changes in the hippocampus have been associated with an increased risk of developing schizophrenia and memory impairments (Cheng and Liu, 2020). Likewise, research has shown that MetS may also play a role in the development of mental and neurological disorders. Several studies have found a strong association between MetS and an increased risk of developing depression, anxiety, and cognitive impairment in adolescence (Moreira et al., 2019; Moradi et al., 2021). Moreover, MetS has been linked to structural brain changes, such as reduced gray matter volume in the prefrontal cortex, hippocampus, and striatum, which are associated with impaired cognitive and emotional functioning (Yau et al., 2012; Etchegoyen et al., 2018).

The concurrence of neuroanatomical changes during adolescence and MetS may further enhance the risk of developing mental and neurological disorders. Studies found that adolescents with MetS had reduced gray matter volume in brain regions associated with cognitive and emotional processing like the prefrontal cortex and the amygdala compared with their counterparts without MetS (Chen et al., 2020; Kim et al., 2020). Likewise, Moreno-Lopez et al. (2016) found that adolescents with MetS showed altered functional connectivity in brain regions associated with cognitive and emotional processing, including the prefrontal cortex and the anterior cingulate cortex.

Resting-state functional connectivity is another measure of brain function that has been investigated in young adults with MetS. Compared with healthy controls, young adults with MetS had reduced functional connectivity between the insula and several brain regions involved in cognitive control and emotional processing. These alterations in functional connectivity may underlie the cognitive deficits and emotional dysregulation commonly observed in people with MetS (Musen et al., 2012; Rashid et al., 2021).

### EE counteracting effects on MetS-associated CNS impairment in adolescence

A potential strategy to mitigate the effects of MetS on brain structure and function is taking advantage of EE. In fact, studies in animals have shown that EE exposure can promote neurogenesis, increase synaptogenesis, and improve cognitive function (Han et al., 2022). In humans, EE has been found to be beneficial for cognitive and emotional functioning in healthy individuals as well as those with neurodegenerative diseases, among other pathologies (Sampedro-Piquero and Begega, 2017; Liew et al., 2022).

Given the potential benefits of EE, it may be a useful adjunctive treatment for adolescents with MetS. Providing opportunities for increased physical activity, social interaction, and cognitive stimulation may help to mitigate the negative effects of MetS on brain structure and function. Additionally, incorporating strategies such as mindfulness training, cognitive-behavioral therapy, and stress reduction techniques may help to address the emotional dysregulation commonly observed in individuals with MetS.

Several studies have investigated the effects of EE on the brain, using running wheels — physical exercise — and have measured cognitive outcomes in animal models of MetS. In adolescence, EE exposure improves cognitive functions like learning, memory, and attention in animal models with MetS-like symptoms (Higarza et al., 2021). Also, EE exposure has been linked to neuroplasticity and neurogenesis in the hippocampus, a brain region important for learning and memory. In animal models of MetS, EE has been shown to increase the number of newly generated neurons in the hippocampus (Ho et al., 2013; Nawaz et al., 2018; Higarza et al., 2021). Moreover, EE exposure during adolescence has been shown to increase the expression of neurotrophins such as BDNF and nerve growth factor (NGF) (Parks et al., 2008). These neurotrophins promote neuroplasticity and are essential for the survival and growth of neurons.

EE exposure also has positive effects on the metabolic profile and insulin sensitivity in animal models of MetS. EE exposure during adolescence has been shown to improve insulin sensitivity and reduce hyperglycemia, and hyperinsulinemia in rats with MetS-like symptoms (Parks et al., 2008). Moreover, EE has been shown to reduce the levels of triglycerides and cholesterol in animal models of MetS (de Souza et al., 2019; Díaz de León-Guerrero et al., 2022). A study by Díaz de León-Guerrero et al. (2022) showed that EE improved insulin sensitivity, reduced adiposity, and decreased blood pressure in obese mice, regardless of performing physical exercise. These findings suggest that EE can improve metabolic health and reduce the risk of developing MetS in animal models.

The beneficial effects of EE on metabolic health have also been observed in human studies. Lee et al. (2012) found that EE reduced body weight and visceral fat, and improved insulin sensitivity, lipid profile, and blood pressure in obese adolescents compared with their non-obese counterparts. Also, in teenagers, EE improved executive functions and self-efficacy (Staiano et al., 2013; Chen et al., 2016).

The mechanisms underlying the beneficial effects of EE on the brain and metabolic profile in MetS are not fully understood. One proposed mechanism is that EE exposure increases the levels of BDNF, a neurotrophin that promotes neuroplasticity and has been implicated in the pathophysiology of MetS (Lee et al., 2014). BDNF also regulates energy homeostasis and insulin sensitivity, suggesting a potential link between EE, BDNF, and MetS (Kleinridders et al., 2014). Different studies have shown that, in adolescents with MetS-related symptoms, EE increased BDNF levels through aerobic and resistance training over months in EE programs (Lee et al., 2014; Walsh et al., 2018).

**Figure 4:**
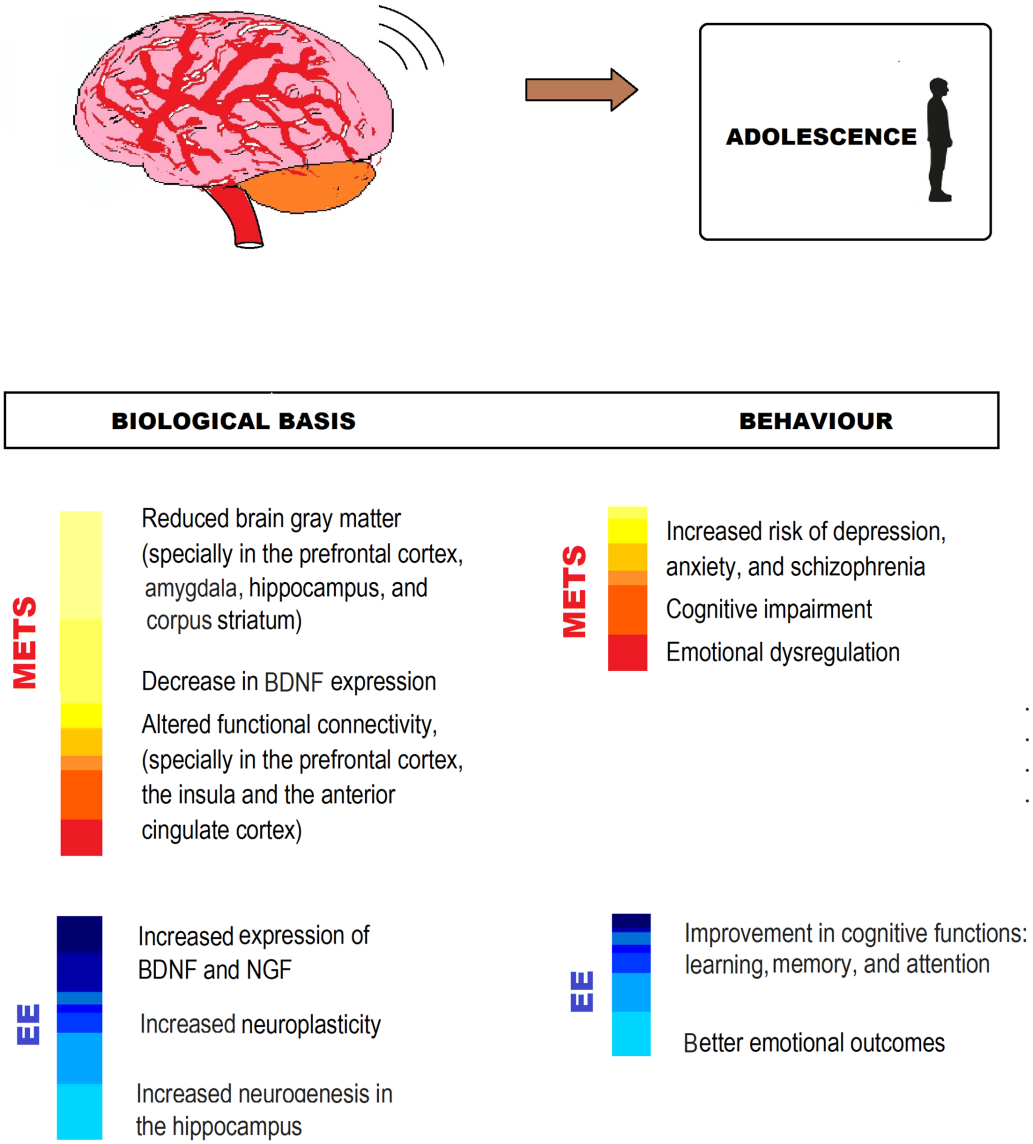
MetS-associated CNS impairment and behavioral changes, and EE counteracting effects in adolescence. The figure shows different ways in which EE exerts neuroprotection from impairment by MetS. IGF, Insuline-like growth factor; BDNF, Brain-derived neurotrophic factor.

Taking into account that adolescence is a critical period for brain development, and environmental factors during this period can have long-lasting effects on the brain and health outcomes, is essential that adolescents can have enough and complex physical, cognitive and social stimulation (Figure 4).

## Adulthood

### CNS development in adulthood

This stage starts immediately after adolescence, at approximately 20 years of age, and during the first phase, known as emerging or early adulthood, the CNS continues on with its development. There are several modifications in the association cortices and the frontal-limbic system, involved in cognitive functions like attention, reward, and executive function, among others. Besides, the white matter continues increasing, likely as a result of the increased myelination with gray matter decrease, and exists a general increase in the prefrontal regulatory function. This is considered a period of vulnerability for psychological impairments and is habitually accompanied by relevant changes in the environment and personal development (Sowell et al., 2003; Taber-Thomas and Pérez-Edgar, 2015). After this stage, at approximately 30 years of age, middle adulthood starts. By this time, while the CNS has already finished its development, changes in response to lifestyle, environment, and other epigenetic factors always take place, along with sustained neurogenesis that influences cerebral plasticity. Slowly, a progressive deterioration of psychobiological and cognitive functions follows that will be intensified in late adulthood or old age (Arias-Carrión et al., 2007).

### Effects of MetS in CNS in adulthood

Concerning the effects of MetS and its components on the CNS in adulthood, a high-saturated fat diet, frequent in Western societies, has been associated with memory impairments, probably due to a decrease in hippocampus volume with worsening in its function (Mestre et al., 2017; Oleson et al., 2017; de Souza et al., 2019; Wahl et al., 2019; Higarza et al., 2021). Also, the excess of saturated fats and added sugar have been related to inflammatory processes in the hypothalamus and leading to insulin resistance and neuronal death, as the hypothalamus is unable to keep homeostasis (Gergerlioglu et al., 2016; Jais and Brüning, 2017).

This diet and other factors contribute to obesity, which is associated with cognitive deficits over lifespan, particularly impairing cognitive flexibility, inhibition, and decision-making (Yau et al., 2014; Haase Alasantro et al., 2021). A high BMI in midlife is a risk factor for cognitive decline and dementia in old age, and obese men are more prone than women to mild cognitive impairment, probably due to estrogenic protection in the latter (Mauvais-Jarvis, 2015; O’Brien et al., 2017). These clinical effects might be caused by the reduction in gray matter in the temporal and frontal lobes, the hypothalamus, and the hippocampus and by cerebral ischemia and hypoperfusion that lead to obesity (O’Brien et al., 2017).

MetS has been often associated not only with deficits in cognitive flexibility and inhibition during adulthood (Yaffe et al., 2004; Falkowski et al., 2014; Haase Alasantro et al., 2021), but also with poor performance in memory, attention, information processing speed, and executive function (Segura et al., 2009; Haase Alasantro et al., 2021). In middle-aged adults with MetS, evidence has been found about a worse performance in memory, verbal fluency, vocabulary, and reasoning in individuals who have been diagnosed with MetS for over 10 years, and a better performance in individuals with non-persistent MetS criteria, which refers to an accumulative effect of MetS in CNS. Also, impairment of executive function and inhibition associated with MetS are likely to contribute to maintaining or worsening the condition in view of the difficulties in keeping a healthy diet or an exercise routine (Haase Alasantro et al., 2021).

MetS in this stage of life has also been related to OS production, mitochondrial dysfunction, cerebral ischemia, and ROS formation, particularly due to hyperglycemia or diabetes, which are silent threats to cognition, and risk factors for neurodegeneration that could lead to Alzheimer’s disease (AD) or dementia (Aoqui et al., 2014; Kim and Feldman, 2015; Etchegoyen et al., 2018). Diabetes also has been connected to other cerebrovascular diseases like hemorrhagic stroke and aneurismal infarcts, among others, that produce many clinical syndromes related to CNS (Nelson et al., 2009). Besides, diabetes produces chronic inflammation, which has been associated with neuronal loss and dysfunction, inhibition of neurogenesis, and worsening of cognitive functions, because of NFkB and proinflammatory cytokines activation that induces cytotoxicity. Obesity also contributes to this inflammation and dysfunction in CNS (Etchegoyen et al., 2018; Karczewski et al., 2022). Chronic inflammation has also been related to schizophrenia and other psychiatric pathologies and cognitive impairment (Marrie et al., 2017; Karczewski et al., 2022).

Not only insulin has an important role in the regulation of glucose metabolism, but is also involved in other functions in the CNS, related to synaptic plasticity and cognitive performance. Insulin resistance (IR) impairs neuronal response to growth factors, leading to neurodegeneration and neuropathy. This is the main reason why diabetes or hyperinsulinemia could be associated with brain degeneration (Lee et al., 2016; Etchegoyen et al., 2018). Recent studies have shown that insulin affects mood and could lead to depression, anhedonia, and behavioral despair due to its actions in the CNS (Moulton et al., 2015; Lee et al., 2016). Both diabetes and depression are associated with HPA axis overactivation (Stetler and Miller, 2011) and deficiency of BDNF, which plays a central role in synaptic connection, neuronal repair, and CNS maturation and plasticity (Eyileten et al., 2017).

### EE counteracting effects on MetS-associated CNS impairment in adulthood

Evidence shows that impoverished living conditions and physical inactivity worsen MetS deleterious consequences in CNS, leading to stronger cognitive impairments. On the contrary, EE has shown beneficial effects on hippocampal neurogenesis, CNS functional connectivity, brain plasticity, synaptogenesis, and BDNF enhancement in animal models, regardless of the practice of physical exercise (Bayat et al., 2015; Narducci et al., 2018; de Souza et al., 2019; Furman et al., 2019), that could counteract cognitive deficits induced by MetS.

In rat models with and without physical exercise, EE resulted in BDNF activation, stimulated angiogenesis, increased CD31 levels, prevented amyloid B accumulation, and induced neurogenesis, increasing neural progenitors’ proliferation in the dentate gyrus (Yu et al., 2014; Gergerlioglu et al., 2016). In obese mice, EE also reduced inflammatory signaling, improved glucose metabolism, and increased anorexigenic signaling in the hypothalamus, restoring the metabolic imbalance caused by a high-fat diet (Díaz de León-Guerrero et al., 2022). In middle aged rats, EE might activate the HPA axis, reducing depression and anxiety partly associated with MetS (Brenes et al., 2016; McMurphy et al., 2018; Queen et al., 2020).

In middle-aged obese adults, exercise, together with dietary changes, improved memory, executive function, psychomotor speed, and learning (Smith et al., 2010; O’Brien et al., 2017). In adults with MetS-related symptoms, EE with physical exercise increased BDNF level, cerebral blood flow, and gray matter density in the hippocampus, the insular cortex, and the cerebellar lobule, and improved diffusivity parameters in surrounding white matter structures and the corpus callosum (Mueller et al., 2015; Domínguez-Sanchéz et al., 2018; Wing and Look AHEAD Research Group, 2021).

Physical activity and EE have shown positive effects on neuroglia, essential for neural plasticity and cognitive functions as increased volume and complexity of astrocytes with enhanced coverage of blood vessels and synapses, improved astrocyte-dependent neurogenesis, and long-term potentiation. EE acted on oligodendrocytes promoting myelination and enhancing microglia proliferation and production of BDNF in mice and rats models both with and without running wheels (Zheng et al., 2019; Leardini-Tristão et al., 2020; Augusto-Oliveira and Verkhratsky, 2021; Verkhratsky et al., 2021). Physical exercise boosts adult neurogenesis, inducing precursor cell proliferation (Kempermann, 2019), and is related to a decrease in proinflammatory cytokines concentration that might help protect the hypothalamus and insulin receptors (Singh et al., 2012).

The hippocampus is a key structure in memory, learning, and emotions, and the evidence about EE changes in the hippocampus is impressive. EE deeply stimulates adult hippocampal neurogenesis, and these new neurons contribute to cognitive performance and affective behavior. In rats, the consumption of a high-fat diet impairs hippocampus-dependent cognitive functions like memory and spatial learning, whereas these functions improve as a result of EE with physical exercise (Gergerlioglu et al., 2016).

The EE through complex activities at the cognitive level, whether work or leisure, produces a slower cognitive deterioration and less expression of clinical symptoms if the subject suffers from AD or dementia in the future, in addition to lowering the probability of its occurrence (Lee et al., 2019; Schoentgen et al., 2020). Likewise, adults who speak more than one language could delay the onset of AD symptoms by 4/5 years compared to those who speak only one language (Bialystok et al., 2014; Vasquez et al., 2014).

**Figure 5:**
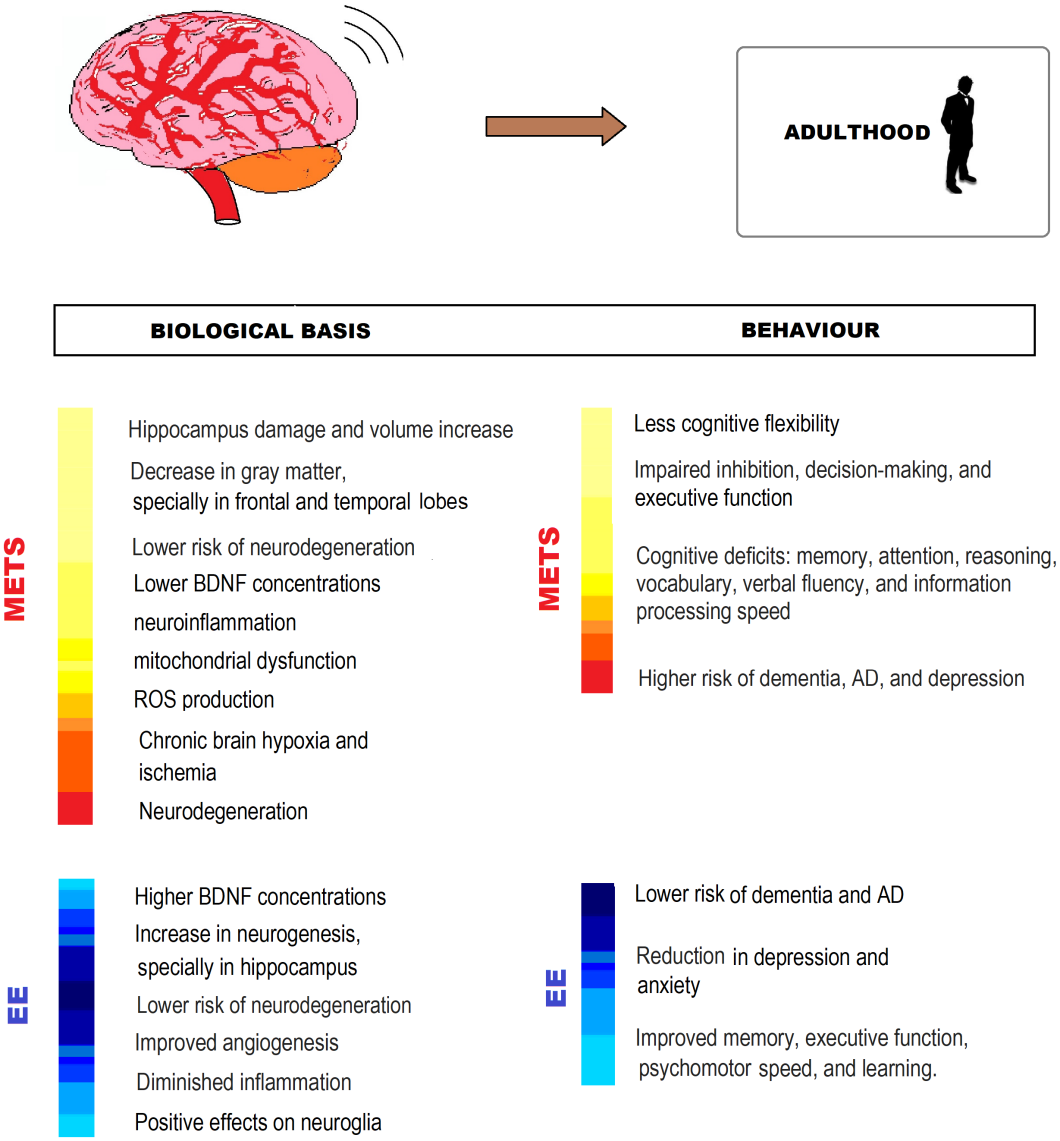
MetS-associated CNS impairment and behavioral changes, and EE counteracting effects in adulthood. The figure shows different ways in which EE exerts neuroprotection from impairment by MetS. BDNF, Brain-derived neurotrophic factor; ROS: reactive oxygen species; AD, Alzheimer’s disease.

EE in early and mid-adulthood has been shown to have beneficial effects not only on people who have developed MetS but also on healthy individuals. In particular, in people at risk of MetS and in people in general, engaged in healthy aging, EE offers a preventive strategy against developing MetS at any time. For this reason, it is relevant that if enrichment did not start in childhood or adulthood, it does at this stage. In this sense, it is extremely important, both at the individual and community levels, to encourage physical exercise, intellectual and leisure activities, and participation in social networks, as an important part of preventive or therapeutic strategies not only in MetS but also to promote healthy adulthood and old age in the general population (Figure 5).

## Old Age

### CNS development in old age

Aging is a multifactorial and complex process that includes the joint effects of genetics, lifetime, behavior, and environment on body organs, systems, and functions, leading to a progressive functional and anatomical decline (Alfaro et al., 2018). This occurs at molecular, cellular, and histological levels in the whole body, but especially at the CNS (Zia et al., 2021). There are several age-related, both functional and structural modification in the CNS, that include decreased reserves of oxygen in the brain (Vasquez et al., 2014), a decrease of about 10–15% of the brain size, especially in the temporal lobe, hippocampus and prefrontal cortex (Vasquez et al., 2014; Alfaro et al., 2018; Wahl et al., 2019) increased neuroinflammation (Guillemot-Legris and Muccioli, 2017), reduced spine density, and diameter, length and branching of dendrites, and reduced synaptic plasticity (Vasquez et al., 2014; Isaev et al., 2019; Queen et al., 2020), a decline in hippocampus neurogenesis, a decrease in brain-derived neurotrophic factor (BDNF) levels and its associated genes (Garrido Vega, 2015; Queen et al., 2020), mitochondrial dysfunction and oxidative damage (Mattson and Arumugam, 2018), among others. Aging also modifies some neurotransmission systems, like the dopaminergic, cholinergic, and glutamatergic circuits (Garrido Vega, 2015).

All these features lead to a loss in the ability of the organism to maintain homeostasis and deficits in the regulation of the hypothalamic–pituitary–adrenal axis (Garrido Vega, 2015). These modifications can cause a decrease in cognitive skills, especially in memory, executive function, and mental processing speed (Vasquez et al., 2014; Alfaro et al., 2018; Wahl et al., 2019; Queen et al., 2020).

Cognitive impairment might be expected in very old adults and minor cognitive modifications may be acceptable during normal aging. However, when cognitive deficits are sustained and relevant, affecting daily routines and performance, they may turn into dementia, Alzheimer’s, or Parkinson’s diseases. By then, the former lifestyle and prevailing environmental conditions become crucial in determining the progress into healthy or unhealthy aging (Alfaro et al., 2018; Wahl et al., 2019; Queen et al., 2020), and/or the severity of disease.

### Effects of MetS in CNS in old age

It has been shown that there is a strong relationship between cardiometabolic health and the risk of dementia. In fact, some important risk factors for this pathology are obesity, diabetes, and MetS (Topiwala et al., 2018). High-fat diets lead to inflammation, hypertension, and insulin resistance, contributing to dementia (Freeman et al., 2014). The reasons for this connection are that a high-fat diet leads to chronic brain hypoxia and ischemia, and excessive microglial activation, contributing to white matter loss and cognitive impairments (Knight et al., 2016; Anjum et al., 2018). These unhealthy diets are associated with reduction and atrophy in hippocampus volume and the resulting memory decline (Mestre et al., 2017; Oleson et al., 2017; Yeomans, 2017). Damage in the hippocampus is related particularly to an increase in proinflammatory-cytokines and hypersensitive microglia, that enhance neuroinflammation. This deleterious effect on the hippocampus can produce a malfunction in the mechanisms related to synaptic plasticity and long-term memory (Wahl et al., 2019).

Sedentarism usually goes hand in hand with MetS, the same as with MetS’ individual components, and is frequent in old adults (Al-Dayyat et al., 2018). This has deleterious consequences on the brain, particularly the reduction of the temporal lobe, which can contribute to memory problems (Siddarth et al., 2018).

Insulin resistance is also a risk factor for cognition impairment, and especially memory, probably because of its worsening of frontal cortex functions, related to a chronic low-grade pro-inflammatory state. This imbalance in the markers of inflammation has been associated with an increased probability of developing dementia because the increased levels of cytokines can cross the brain–blood barrier (BBB), likely to some extent defective during aging, and produce chronic inflammation, resulting in neurodegenerative processes (Pugazhenthi, 2017; Arshad et al., 2018; Atti et al., 2019).

Metabolic syndrome has been shown to be a key risk factor in the development of Alzheimer’s disease (AD) (Tolppanen et al., 2014). Hyperglycemia and obesity are associated with a greater risk of AD, because of their contribution to chronic inflammation, oxidative stress, and accumulation of amyloid plaques in the brain that are implicated in AD etiology (Pugazhenthi, 2017; Wahl et al., 2019; Chukwuma, 2023). MetS has been related to vascular dementia development because this cluster of clinical features raises the risk of silent brain infarction and recurrent stroke, and stroke volume is critical in this kind of dementia (Li et al., 2017; Atti et al., 2019). Cerebral microinfarcts associated with MetS are thought to contribute independently to cognitive impairment due to oxidative stress, hypoperfusion, and inflammation (Pugazhenthi, 2017).

Bearing this in mind, MetS can add to and even potentiate cognitive impairment or dementia development and progression. Accordingly, healthy lifestyle changes that ameliorate MetS-associated cerebrovascular compromise also help reduce mental and cognitive risks, improving performance.

### EE counteracting effects on MetS-associated CNS impairment in old age

The EE model can reduce or prevent age-related functional worsening, particularly MetS deleterious neurological consequences. Both in animal and human studies, EE has been largely shown as a proven strategy to increase neurogenesis, dendritic spine number and complexity, synaptic plasticity, and cognitive flexibility, among others (Consorti et al., 2019; Gelfo, 2019). For these reasons, it has been used to control neurodegeneration in Alzheimer’s, Parkinson’s, and Huntington’s diseases (Vasquez et al., 2014; Nelson and Tabet, 2015; Wassouf and Schulze-Hentrich, 2019; Queen et al., 2020).

EE has a central role in activating the hypothalamic-sympathoneural-adipocyte (HSA) axis. Physical, cognitive, and social stimuli induce BDNF expression in the hypothalamus, boosting the sympathetic input into the adipose tissue, which downregulates leptin, resulting in an antiobesity phenotype. EE-induced HSA activation is key in counteracting MetS-related features and MetS impact on the brain. EE with running wheels reduced adiposity, hyperglycemia, and dyslipidemia in different mice models (McMurphy et al., 2018; Huang et al., 2020). Short-term EE exposure results in the upregulation of hypothalamic BDNF, improved glycemic control, and decreased fat mass in old mice with MetS (Queen et al., 2020). Physical activity restores high levels of insulin-growth factor (IGF), which raises BDNF concentration and increases neurogenesis, likely improving cognitive outcomes in rats (Carro et al., 2000). BDNF availability has a direct relation with neuron proliferation in the hippocampus and affects synaptic plasticity and neurotransmitter release, including cholinergic and dopaminergic systems, usually affected by this life stage (Vaynman et al., 2004; Zia et al., 2021).

Long-term EE with physical exercise increased microglia cell number and ramification, improved HSA axis regulation, and reduced neuroinflammation markers in mice. Improved learning parameters, exploratory behavior, and locomotor activity, and decreased lipofuscin load in the dentate gyrus were also reported, indicating a decrease in nonspecific age-dependent degeneration (Kempermann et al., 2002; Huang et al., 2020; Queen et al., 2020). Animal and human studies have found a positive effect of EE on neurogenesis in the hippocampus, related to a protective effect on memory (Richards and Frankland, 2017).

Physical activity, a central component of EE neuroprotection in MetS, improves cognitive skills, especially memory and executive function, often impaired by MetS, and reduces the risk of developing neurodegeneration and dementia in old adults (Paillard et al., 2015; Hamer et al., 2018; Leon and Woo, 2018; Zotcheva et al., 2018; Engeroff et al., 2019). These effects appear related to an increase in BDNF production and synaptogenesis because of increased neuronal excitability (Loprinzi et al., 2018; Sungkarat et al., 2018), an increase in brain volume, in the hippocampus in particular (Niemann et al., 2014), a decrease in oxidative stress (Rytz et al., 2020) and an improvement in neurogenesis, synaptic plasticity, brain flow, and cortical inter-hemispheric connectivity, that have been seen in several studies in old adults (Vasquez et al., 2014; Paillard et al., 2015; Leon and Woo, 2018; Zia et al., 2021). In this population, physical activity also reduces cardiovascular risk (Hamer et al., 2018), excess body, and resistance to diet-induced obesity (McMurphy et al., 2018). Conversely, poor physical activity has been associated with the prevalence, development, and pathogenesis of MetS (Yamaoka and Tango, 2012; Rytz et al., 2020).

Cognitive stimulation is another component of EE, which is administered through exposure to different textures, tunnels, and nesting materials, among others, to foster exploration in animal models. In humans, EE comprises learning new languages or skills, solving puzzles and games, reading, creative work, etc. Many studies have shown that, in the elder, these elements foster cognitive performance, especially memory and executive functions, often the most affected in elders with MetS. Likewise, there is evidence of a decrease in the risk of developing dementia and overall cognitive deterioration in response to EE exposure strategies (Bialystok et al., 2014; Vasquez et al., 2014). Multisensory stimulation models virtually like those used in animals have been tried in people with disabilities, Alzheimer’s disease, and dementia, through various tactile, vibratory, olfactory, visual, and auditory stimuli, etc. using music, water beds, vibrating mattresses, just as a few examples and positive effects have been observed at the behavioral and emotional levels in particular. So far, there is still insufficient evidence of their neurobiological and cognitive consequences (Van Weert and Bensing, 2009; Rodríguez and Llauradó, 2010).

The increase in social networking and activities, another component of EE, can slow down age-related cognitive impairment and protect against dementia both in old animals and humans (Xu et al., 2015; Wahl et al., 2019; Schoentgen et al., 2020). In rodents, improved memory associated with neuronal protection in the hippocampus has been reported, the same as better spatial learning (Pereda-Pérez et al., 2013). In older people, social interaction produces better performance on tests related to executive function, memory, and intelligence, also expanding cognitive reserve, while the opposite is found in older adults with reduced social contacts (Seeman et al., 2011; Vasquez et al., 2014).

**Figure 6:**
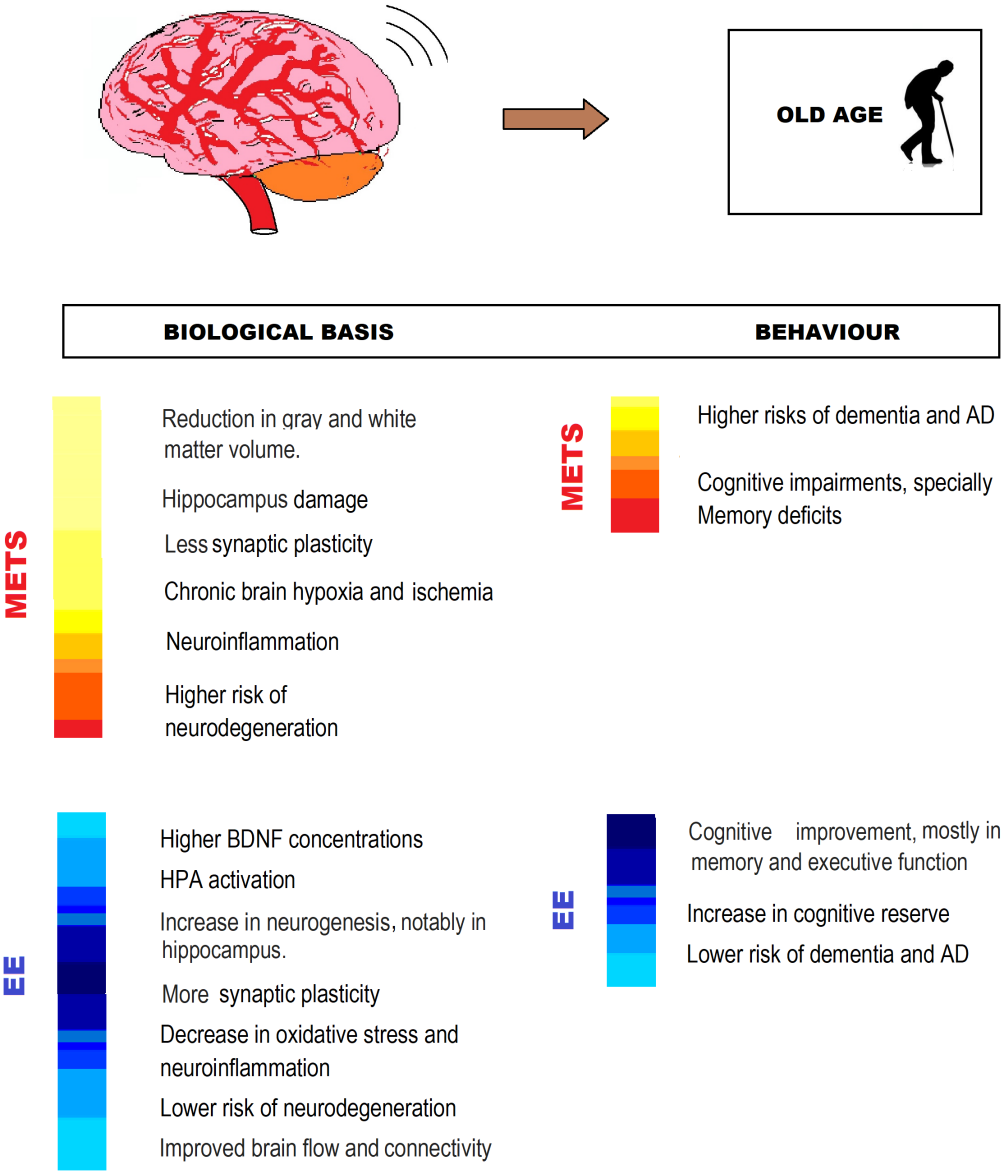
MetS-associated CNS impairment and behavioral changes, and EE counteracting effects in old age. The figure shows different ways in which EE exerts neuroprotection from impairment by MetS. IGF: Insulin-like growth factor; BDNF: Brain-derived neurotrophic factor; AD, Alzheimer’s disease; HPA, hypothalamic–pituitary–adrenal axis.

Currently, there are EE programs for older adults that, while not specifically intended for MetS, seem promising for the neurological deterioration associated with MetS. The Fit Brains Program offers combined physical and cognitive training (Ten Brinke et al., 2018). The proposal of Eggenberger et al. (2015) includes varied physical training combined with training in executive functions, episodic memory, and processing speed. In the same line, Best et al.’s (2018) contribution to chronic stroke elders includes physical, cognitive, and social stimulation through the Lumosity program (Mendes et al., 2022) (Figure 6).

## Discussion and future perspectives

The human Central Nervous System undergoes development over the course of an individual’s life, causing modifications in cognitive abilities from childhood to advanced age. As a result, investigations on cognition and its underlying factors will be enhanced by utilizing lifespan methodologies that acknowledge the progression of development, and hence can strategize preventive measures from early stages of life, identifying the risk factors for regular neurodevelopment right from the outset.

It is imperative to determine the effects that MetS has on the CNS during each stage of development, as ontogeny shapes individual characteristics, and environmental factors have a crucial influence. It is important to recognize that CNS development is not only dependent on genetic programming, but also on epigenetic changes, environmental factors, and lifestyle modifications. Therefore, it is crucial to identify these alterations and implement appropriate measures to address them.

It is of great importance to examine thoroughly the factors that increase the risk and the neurobiological mechanisms that give rise to cognitive decline associated with MetS, as well as the fundamental aspects of the neuroprotective effects provided by EE. In this regard, it is crucial to highlight the necessity of further investigating the influence of the hypothalamic–pituitary–adrenal (HPA) axis and BDNF on MetS and its potential amelioration through EE.

It is worth noting that various experiments have been conducted on animals, particularly rodents, using EE housing, and attempts have been made to apply this paradigm to humans. Despite the methodological limitations of replicating EE studies in humans, the integration of the different components of the EE model in animals has yielded positive results, and its application in humans through sensory, cognitive, and physical stimulation programs has proven useful for both healthy and ill individuals. However, there have been relatively few attempts to apply this approach to MetS and its associated conditions, especially in childhood, where physical exercise is typically the only component included, with cognitive stimulation included at most. EE models that include sensory, cognitive, social, and physical stimulation factors have only been found in old age, with few programs incorporating more than two types of stimulation, and none specifically for MetS. If the empirical evidence indicates a positive impact of stimulation through the isolated components of the EE model in humans, an integrated investigation of all four components may yield significant findings.

As we delve further into the range of potential applications and the intricacies of established mechanisms involved in laboratory EE models, our capacity to proficiently implement their principles in human subjects will be enhanced. The outcomes obtained and anticipated from such endeavors, alongside public health campaigns and epidemiological assessment, have considerable prospects for enhancing human healthspan.

It is important to investigate animal models of MetS and EE in order to better understand their transferability to humans and the critical stages at which to apply them. The impact of EE exposure on individuals at different ages and for different durations is also poorly understood. Therefore, it is essential to investigate whether EE programs implemented during early life are as effective as those in old age and to assess both their short-term and long-term effects. These studies may have significant implications for applied and therapeutic purposes, as they would help us comprehend brain plasticity and neurodevelopment across the lifespan and enable the development of intervention plans based on EE’s protective factors for MetS.

The promotion of applied research based on the relationship between MetS and the EE model from the field of neuroscience is of great significance, as it can help increase public awareness and attention toward aspects such as education, physical and cognitive leisure activities, social spaces, and intellectual job opportunities that enhance human capacities and well-being at all stages of development. Such research can also provide scientific evidence for the development of programs and interventions that are beneficial for the prevention and treatment of MetS and related conditions.

It would be advantageous to integrate new technologies into the EE. The use of the Lumosity application for cognitive stimulation in older adults has been noted in the review, or the S&G program for children, and Nintendo Wii Active games for teenagers. The use of applications, chats and gamification could be an accessible and enjoyable stimulation to introduce to younger individuals, particularly during the stages of puberty, adolescence, and young adulthood. Therefore, more research should be conducted to assess the potential cognitive benefits of new technologies.

Given the significant increase in lifespan over the past decades, one of the fundamental goals of EE interventions in MetS should be extending healthspan — is the period of life spent in good health, free from chronic diseases or disabilities. When there is a mismatch between lifespan and healthspan, satisfaction and overall functionality decrease, while pain and discomfort increase. This affects both the individual and social levels, increasing healthcare costs and reducing productivity. All stages of lifespan need to be individually considered, and the cycle as a whole, to turn it into a healthspan. As vital stages progress, both cognitive functions and health progressively decline, making these interventions even more critical. EE is a powerful ally that can be carried out economically and personally and should be given priority over alternative invasive interventions or those with potential adverse effects like overmedication or excessive pharmacotherapy.

## Author contributions

TK: conceptualization, writing original draft, and updated research. CM, NT-U: writing original draft and updated research. RK-F: bibliographic research and figure design. FC: supervision and funding acquisition. MO-L: supervision, writing revision, conceptual, structural, and language editing and proofreading. All authors contributed to the article and approved the submitted version.

## Funding

PS1 UAI-CONICET COFECYT-MINCYT 2022.

## Conflict of interest

The authors declare that the research was conducted in the absence of any commercial or financial relationships that could be construed as a potential conflict of interest.

## Publisher’s note

All claims expressed in this article are solely those of the authors and do not necessarily represent those of their affiliated organizations, or those of the publisher, the editors and the reviewers. Any product that may be evaluated in this article, or claim that may be made by its manufacturer, is not guaranteed or endorsed by the publisher.
