# Corrigendum: Neuroprotection in metabolic syndrome by environmental enrichment. A lifespan perspective

**DOI:** 10.3389/fnins.2023.1279346

**Published:** 2023-09-08

**Authors:** Tamara Kobiec, Claudia Mardaraz, Nicolás Toro-Urrego, Rodolfo Kölliker-Frers, Francisco Capani, Matilde Otero-Losada

**Affiliations:** ^1^Facultad de Psicología, Centro de Investigaciones en Psicología y Psicopedagogía, Pontificia Universidad Católica Argentina, Buenos Aires, Argentina; ^2^Centro de Altos Estudios en Ciencias Humanas y de la Salud, Universidad Abierta Interamericana, Consejo Nacional de Investigaciones Científicas y Técnicas, Buenos Aires, Argentina; ^3^Facultad de Ciencias de la Salud, Instituto de Ciencias Biomédicas, Universidad Autónoma de Chile, Santiago, Chile

**Keywords:** metabolic syndrome, environmental enrichment, neuroprotection, neurodevelopment, lifespan, healthspan

In the published article, there was an error in original affiliations 3, 4. Affiliations “^3^Facultad de Psicología y Psicopedagogía, Pontificia Universidad Católica Argentina, Buenos Aires, Argentina, ^4^Departamento de Biología, Universidad Argentina John F. Kennedy, Buenos Aires, Argentina”, they were incorrectly attributed to Francisco Capani, but do not belong to any author.

The authors apologize for this error and state that this does not change the scientific conclusions of the article in any way. The original article has been updated.

